# RGB Color-Discriminable Photonic Synapse for Neuromorphic Vision System

**DOI:** 10.1007/s40820-024-01579-y

**Published:** 2024-11-29

**Authors:** Bum Ho Jeong, Jaewon Lee, Miju Ku, Jongmin Lee, Dohyung Kim, Seokhyun Ham, Kyu-Tae Lee, Young-Beom Kim, Hui Joon Park

**Affiliations:** 1 Department of Organic and Nano Engineering & Human-Tech Convergence Program, Hanyang University, Seoul, 04763 Korea; 2 Department of Semiconductor Engineering, Hanyang University, Seoul, 04763 Korea; 3 Department of Mechanical Engineering, Hanyang University, Seoul, 04763 Korea; 4 Department of Physics, Inha University, Incheon, 22212 Korea

**Keywords:** Organic field-effect transistor, Photonic synapse, Excited-state dipole moment, RGB color discrimination, Neuromorphic visual system

## Abstract

To emulate the functionality of the human retina and achieve a neuromorphic visual system, the development of a photonic synapse capable of multispectral color discrimination is of paramount importance. However, attaining robust color discrimination across a wide intensity range, even irrespective of medium limitations in the channel layer, poses a significant challenge. Here, we propose an approach that can bestow the color-discriminating synaptic functionality upon a three-terminal transistor flash memory even with enhanced discriminating capabilities. By incorporating the strong induced dipole moment effect at the excitation, modulated by the wavelength of the incident light, into the floating gate, we achieve outstanding RGB color-discriminating synaptic functionality within a remarkable intensity range spanning from 0.05 to 40 mW cm^−2^. This approach is not restricted to a specific medium in the channel layer, thereby enhancing its applicability. The effectiveness of this color-discriminating synaptic functionality is demonstrated through visual pre-processing of a photonic synapse array, involving the differentiation of RGB channels and the enhancement of image contrast with noise reduction. Consequently, a convolutional neural network can achieve an impressive inference accuracy of over 94% for Canadian-Institute-For-Advanced-Research-10 colorful image recognition task after the pre-processing. Our proposed approach offers a promising solution for achieving robust and versatile RGB color discrimination in photonic synapses, enabling significant advancements in artificial visual systems.

**Supplementary Information:**

The online version contains supplementary material available at 10.1007/s40820-024-01579-y.

## Highlights

- Photonic synapse capable of multispectral color discrimination is demonstrated.
- Strong excited-state dipoles enable remarkable discrimination intensity (0.05–40 mW cm^-2^).
- This approach is not restricted to a specific medium in the channel layer, and convolutional neural network with synapses array achieves over 94% inference accuracy for Canadian-Institute-For-Advanced-Research-10 images.

**Supplementary Information:**

The online version contains supplementary material available at 10.1007/s40820-024-01579-y.

## Graphical abstract

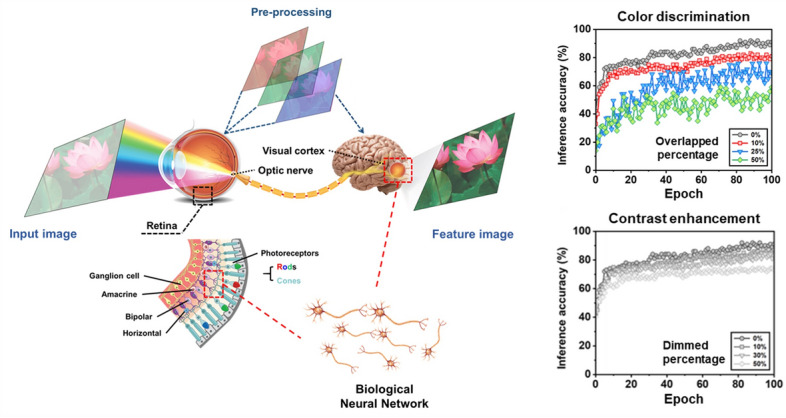

## Introduction

Human body perceives over 80% of information from the intricate external world through its visual sensory system [1, 2]. The retina, located within the human eyes, plays a crucial role in this process by detecting and pre-processing light signals in the early stages of visual perception, which precedes the complex, high-level signal processing performed by the visual cortex of the brain. The retina acts as a vital intermediary between the external world and the visual interpretation of the brain [3, 4].

In the process of detection, visual input is converted into electrical signals by photoreceptors, which are then transmitted to the brain. As part of the pre-processing stage, the retina fulfills critical functions such as filtering, amplifying, adaptation, and memorization. These functions are carried out by the synapses that connect various types of neurons, such as photoreceptor, horizontal, bipolar, amacrine, and ganglion cells, which are interconnected to form a hierarchical biostructure within the retina [5]. This pre-processing is crucial for reducing redundant visual data, thereby improving the efficiency of complex data processing in the brain, including image recognition, learning, and interpretation [6]. Therefore, to successfully replicate the functionalities of the retina and create an efficient artificial visual system, it becomes imperative to demonstrate optoelectronic devices that possess the capability converting light signals into electrical signals while containing synaptic plasticity for pre-image processing.

An essential characteristic of the retina enabling these functionalities is its ability to recognize color information. Therefore, optoelectronic synapse devices intended to emulate the retina need to possess multispectral color-discriminating capabilities to effectively realize an artificial visual system. This particular feature offers several additional benefits to the system, such as reducing the size and complexity of individual pixels, by eliminating the need for optical filters and the corresponding intricate circuitry.

Early-stage works have focused on integrating a photodetector with a synaptic device, resulting in complex circuitry [7, 8]. To address these limitations, various strategies have been proposed, such as utilizing specific medium for absorbing photon [9–13], generating trapped charge [11, 13, 14], controlling defect density [10, 12], adjusting doping [15], and changing valence states [16]. However, achieving color discrimination characteristics proves challenging as it requires specific material combinations as mediums, thus limiting its practical application. An alternative approach entails utilizing a three-terminal phototransistor equipped with mixed quantum dots (QDs) capable of absorbing light across multiple wavelengths [9]. This configuration demonstrates a color discrimination capability; however, it is within a restricted intensity range of 20 mW cm^−2^. Additionally, QD-modified synaptic transistor has been employed to demonstrate luminance-based color-to-gray conversion [17]. Meanwhile, a photonic synapse, capable of converting mixed colors into spike trains with different frequencies, has been reported [18]. Recently, several studies have explored the incorporation of light-absorbing materials as floating gates, rather than channel layers, in a three-terminal transistor flash memory architecture [19–24]. However, effectively achieving robust color discrimination capabilities through these approaches continue to pose a challenge, as the existing photo-programming window does not offer adequate scope for comprehensive color discrimination and it is not adjustable depending on the wavelength of the light.

In this work, we propose an approach that can provide a superior color-discriminating synaptic functionality to a three-terminal transistor flash memory architecture. Our method introduces a floating gate that not only expands the photo-programming window but also enables wavelength-dependent adjustability. This is accomplished by harnessing the induced dipole effect at the excitation, combined with the trapped photo-carrier effect within the floating gate, to modify the threshold voltage (*V*_th_) of the device. The intensity of the induced dipole moment effect can be precisely controlled according to the wavelength of the incident light. As a result, our approach achieves superior color discrimination functionality with a wavelength-dependent adjustable photo-programming window, operating within an impressive intensity range of 40 mW cm^−2^. Given that this photo-functionality is achieved through the utilization of the floating gate, our approach is not restricted to a specific medium in the channel layer, thereby possessing universality that can broaden its applicability. We have validated this concept with various devices employing different channel materials.

The enhanced induced dipole effect under light illumination, generated within a floating gate, can be achieved by utilizing asymmetric molecules that exhibit a high intrinsic dipole moment during excitation even with an extended lifetime. Importantly, the degree of the induced dipole moment effect depends on the density of excited molecules, which is influenced by their spectral absorbance and excited-state lifetime. This spectral dependency results in a remarkable color-discriminating capability. We confirm that the array of the photonic synapses, exhibiting the efficacy of this color-discriminating functionality, maximizes the inference accuracy of a convolutional neural network (CNN) in colorful image recognition tasks through its visual pre-processing. This pre-processing involves the differentiation of RGB channels and the enhancement of image contrast with noise reduction.

## Experimental

### Materials

2,7-dioctyl[1]benzothieno[3,2-b][1]benzothiophene (C8-BTBT) was purchased from Sigma-Aldrich (≥ 99%, HPLC). 2,3,5,6-tetrafluoro-tetracyanoquinodimethane (F4-TCNQ) was purchased from Lumtec. Poly[methyl methacrylate] (PMMA) was purchased from Sigma-Aldrich (Average Mw ~ 120,000 by GPC). Chlorobenzene (CB, anhydrous, 99.8%) and 2-butaneone (anhydrous, 99%) were purchased from Sigma-Aldrich. The general synthesis procedures for the organic molecules used in the floating gate are detailed in Note S1.

### Organic Semiconductor Solution Preparation

For the high-orientational uniform film, small molecular semiconductor C8-BTBT (5 mg mL^−1^ in CB) and insulating polymer PMMA (2.5 mg mL^−1^ in CB) solutions were mixed at a weight ratio of 2:1. Additionally, for the high-mobility and low-threshold-voltage operation of organic field-effect transistor (OFET), the mixed solution was doped with F4-TCNQ solution (dissolved in 2-butaneone) at a concentration of 1.0 wt% related to the C8-BTBT. After then, the blended solution was further stirred for one day to mix completely.

### Flash Memory Device Fabrication

Synaptic device was fabricated with a bottom-gate top-contact geometry. A heavily doped p-type Si/SiO_2_ substrate (SiO_2_, 300 nm) was sequentially cleaned by acetone and 2-propanol for 0.5 h in the ultrasonic processor. Then, the substrate was rinsed by distilled water (DI water), fully dried by N_2_ blowing, and then oven-dried for 2 h. Organic floating gate thin films (10 nm) were prepared by spin coating their solutions (5 mg mL^−1^ in CB) on the gate dielectric layer (4000 rpm, 30 s), and annealed (room temperature, 30 min) in a glovebox. And then, for the charge tunneling layer, Al_2_O_3_ layer (target thickness: 30 nm) was deposited via atomic layer deposition (ALD) on the organic thin-film floating gate layer. The trimethylaluminum (TMA, (CH_3_)_3_Al) and water were used as a precursor and a reactant source for Al_2_O_3_, respectively. The deposition process temperature of the source/reactant containers was room temperature, and the substrate chamber temperature was 150 °C. During the deposition process, TMA precursor was injected into the reactant chamber for 0.5 s, and the injection time was pulsed for 1 s for the water. The growth rate of Al_2_O_3_ was 1.15 Å/cycle. After ALD deposition process, the C8-BTBT layer with the thickness of around 15 nm was spin-coated on the Al_2_O_3_ layer at 2000 rpm for 40 s, and then annealed at room temperature for 40 min as a semiconductor channel. 40 nm Au was thermally evaporated in a high vacuum system (under a vacuum of 2.0 × 10^−6^ Torr at a deposition rate of 0.1 Å s^−1^) through a shadow mask for top-contact electrodes. For the fabrication of the synapse array, a Si/SiO_2_ substrate was used as the gate and dielectric layer. Following the same procedures as for the single-device fabrication, Au source and drain electrodes were deposited via thermal evaporation to form a 16 × 16 synapse array. This defined the channel length (*L*) and width (*W*) of the fabricated devices as 100 and 1500 μm, respectively.

### Characterization

The cross-sectional image of the device and the thickness of each layer were obtained using a scanning electron microscope (FE-SEM, S-4800, Hitachi). A UV–Vis spectrometer (UV-2600, SCINCO) was utilized for measuring the optical absorbance spectra of the films in the range of 300–800 nm. The steady-state photoluminescence (PL) and time-resolved photoluminescence (TRPL) spectra were recorded using PL spectrophotometer (FlouTime 300) with a detection wavelength range of 550–850 nm. The excitation light sources were 266 nm (DNH and DN) and 405 nm (CH-M and C-M). The values of work function (WF) and valence band maximum (VBM) of prepared thin films were determined through ultraviolet photoemission spectroscopy (UPS, XPS-Theta Probe, Thermo Fisher Scientific Co.) with He I (21.2 eV) as the excitation UV source. The in situ surface potential measurements were taken on AFM (NX20, Parksystems) in ambient conditions at room temperature. All the electrical properties and synaptic characteristics of devices were measured in ambient condition using a semiconductor parameter analyzer (Keithley 2636B) connected with a probe station in dark shield box. Monochromatic light-emitting diodes with wavelengths peaked at 450 nm (B), 525 nm (G), and 630 nm (R), respectively, were used to generate optical stimulus, which were controlled to offer fixed wavelength and different intensities by a pulse generator (SP-300 model potentiostat, BioLogic).

### Neural Network Simulation

In the feed-forward process of CNN architecture, two main parts of operations are performed: feature extraction and classification. In the feature extraction phase, pre-processed color images, of which each pixel is segregated into three RGB color channels, are convoluted with kernels. This process leads to the generation of the feature maps, which are subsequently downsized through the pooling layer. The Canadian-Institute-For-Advanced-Research-10 (CIFAR-10) color images and convolutional kernels consists of 32 × 32 pixels and 3 × 3 pixels, respectively. Each pixel of the input images and the kernel is represented as a voltage (*V*) and synaptic weight (*W*). The outcomes of convolution operation ($I=W\times V$) are expressed in the feature map as the current (*I*), are transformed to the voltage signal by the ReLU activation function, and then passed to the pooling layer to reduce the size of the map. The cycle is iterated three times in succession. Thus, the dimensions of the kernels are 3 (the number of channels) × 3 (kernel height) × 3 (kernel width) × 128 (the number of kernels), 128 × 3 × 3 × 256, and 256 × 3 × 3 × 512 for 1st, 2nd, and 3rd convolution layer, respectively. After each round of pooling, the dimensions of the feature maps are downsized from 32 × 32, 16 × 16, and 8 × 8 to 16 × 16, 8 × 8, and 4 × 4. At the end of final pooling layer, the voltage signals are flattened to a 1 × 8,192 array and transferred to the input layer of the fully connected layers (FCLs), composed of 8,192 input neurons, 1,024 hidden neurons, and 10 output neurons. Between input and hidden neurons, the current values are obtained from the voltage signals through the weighted-sum operation ($I=\sum W\times V$) with the synaptic weights [25]. The derived current signals are converted into the voltage signals by the ReLU activation function and added to the hidden layer as input signals. Subsequently, these voltage signals are utilized to produce the current at the output layer through the weighted-sum operation with the synaptic weights connecting the hidden and output neurons. After the feed-forward process, the backpropagation procedure is performed by comparing output layer value (*V*_o_) and label value (*K*). Synaptic weights are then updated from output layer to the first convolution layer by minimizing the error ($\delta =K-{V}_{\text{o}}$) between output and label values. According to Google Colab, the training and recognition time for a single image takes 2.7 s per iteration and 135 s per epoch which are competitive compared to other study [26]. For reference, one epoch is comprised of 50 iterations.

### NeuroSim as Supporting Module for Neural Network

DNN + NeuroSim framework, wrapped by Python, is used for neural networks (NNs) as a supporting module to emulate the deep neural networks (DNN) inference performance or on-chip training performance on the hardware accelerator based on in-memory computing architectures. At the run-time of NNs, the simulator iteratively performs feed forward (FF) and backpropagation (BP), which contains a series of weighted sum and weight update operations, respectively. For online learning, the simulated weight is adjusted by nonlinear coefficients and pulse values extracted from actual measurements to match the calculated software weight value, and the feed-forward and error computation are done in the transposable synaptic arrays, while weight gradient computation is done in the weight gradient computational units. The measured synaptic characteristics of the DNH-integrated transistor are used as synaptic weight. Since the experimentally measured LTP and LTD characteristics of the photonic synapses are not ideally linear, in the simulated synapse, we use the device model to extract the nonlinearity parameters of the conductance update behaviors as described in Note S2. Additionally, we set the batch sizes to be 60 and conduct 100 epochs for the optimal simulation.

## Results and Discussion

### Operation Principle of Photonic Synapse

**Fig. 1:**
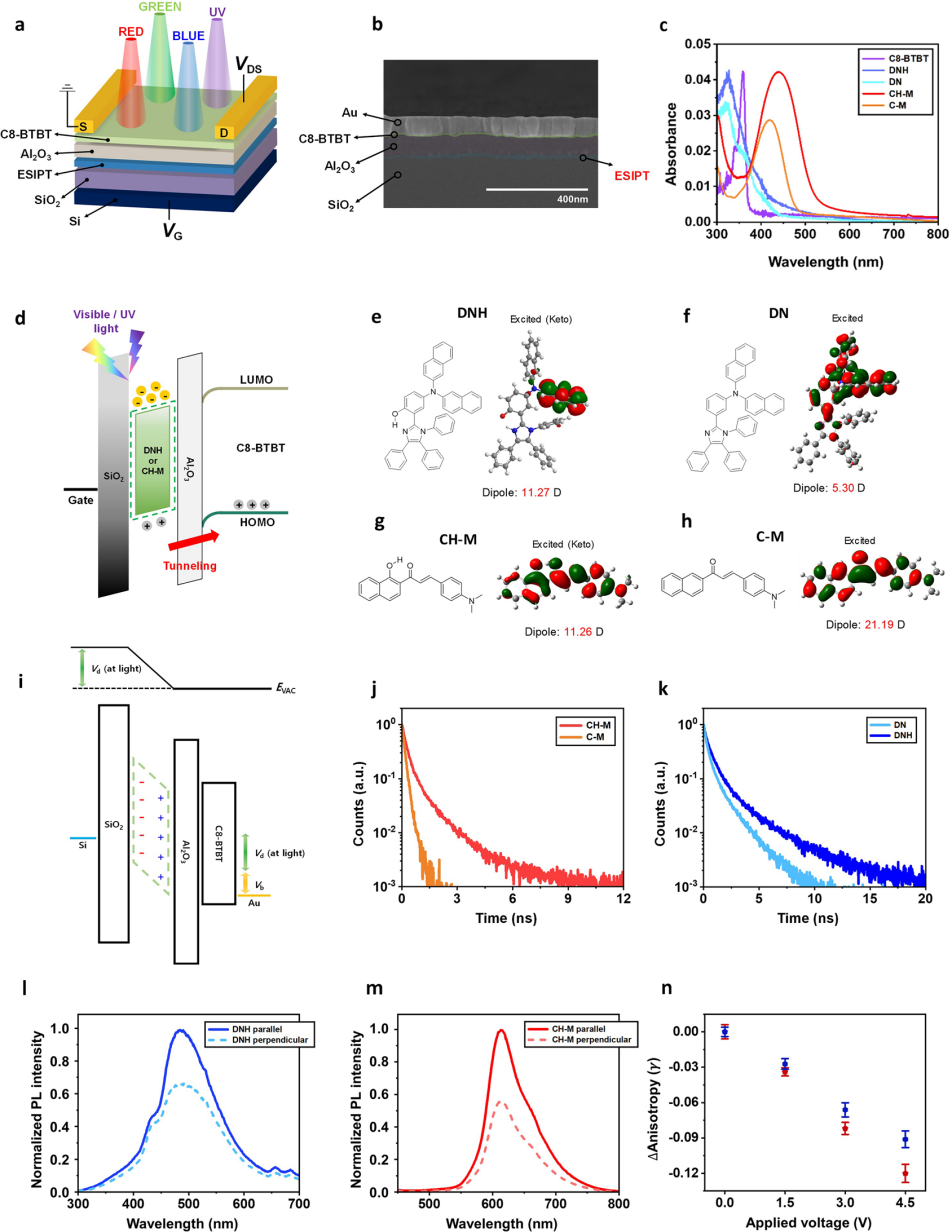
**a** 3D schematic illustration of photonic synapse transistor with flash memory architecture in a bottom-gate top-contact configuration. **b** Cross-sectional SEM image of the photonic synapse device (Si/SiO_2_/ESIPT floating gate/Al_2_O_3_/C8-BTBT/Au). **c** UV–Vis absorption spectra of the organic semiconductor thin films casted on quartz. **d** Energy diagram of photonic synapse transistor in photo-programming mode under light illumination. **e-h** Chemical structures of the light-responsive organic semiconductors and their orbitals in keto form at the excitation (K*) obtained by DFT: (**e**) DNH (ESIPT-active, UV-responsive molecule), (**f**) DN (non-ESIPT, UV-responsive molecule), (**g**) CH-M, ESIPT-active, visible light-responsive molecule, and (**h**) C-M (non-ESIPT, visible light-responsive molecule). Theoretically calculated dipole moment values are added to the figures. **i** Flat-band energy diagram of photonic synapse transistor with induced dipole moments under light illumination. **j, k** Time-resolved photoluminescence (TRPL) decay curves of organic semiconductor thin films casted on quartz: (**j**) CH-M and C-M at 405 nm wavelength excitation. (**k**) DNH and DN at 266 nm wavelength excitation. **l, m** PL anisotropy of ESPIT-active organic thin films: (**l**) DNH (at 266 nm wavelength excitation) and (**m**) CH-M (at 405 nm wavelength excitation). **n** PL anisotropy changes of organic thin films depending on the applied electric field (blue: DNH, red: CH-M)

Figure 1a depicts the device structure of a photonic synapse, which incorporates various light-responsive asymmetric organic layers exhibiting strong dipole moments upon excitation and an extended excited-state lifetime through the excited-state intramolecular proton transfer (ESIPT) process, which leads to an enhanced built-in electric field and an increased number of photoelectrons within the floating gate—details of which will be discussed further. 2,7-dioctyl[1]benzothieno[3,2-b][1]benzothiophene (C8-BTBT) organic semiconductor, exhibiting p-type characteristics, is utilized as a channel layer due to its high stability and mobility [27]. The self-organized properties of C8-BTBT, derived from its own molecular structure, induce the formation of highly ordered crystalline films. This structural orientation minimizes defects in the channel layer, contributing to the high electronic performance and stability of the device [28]. Heavily doped silicon (Si) is employed as the gate electrode. SiO_2_ and Al_2_O_3_ serve as blocking and tunneling layers, and p-type Si is utilized as the gate to avoid the need for a high threshold voltage (*V*_th_) in the accumulation mode [29–31]. A cross-sectional scanning electron microscope (SEM) image of the device in a bottom-gate top-contact configuration is represented in Fig. 1b. The investigation initially focuses on the photonic synapse employing C8-BTBT as the channel layer, serving as a model system. Subsequently, devices featuring alternative channel layers, such as poly(3-hexylthiophene) (P3HT) and polythieno[3,4-b]-thiophene-co-benzodithiophene (PTB7), are also studied to establish the universality of our approach.

**Fig. 2:**
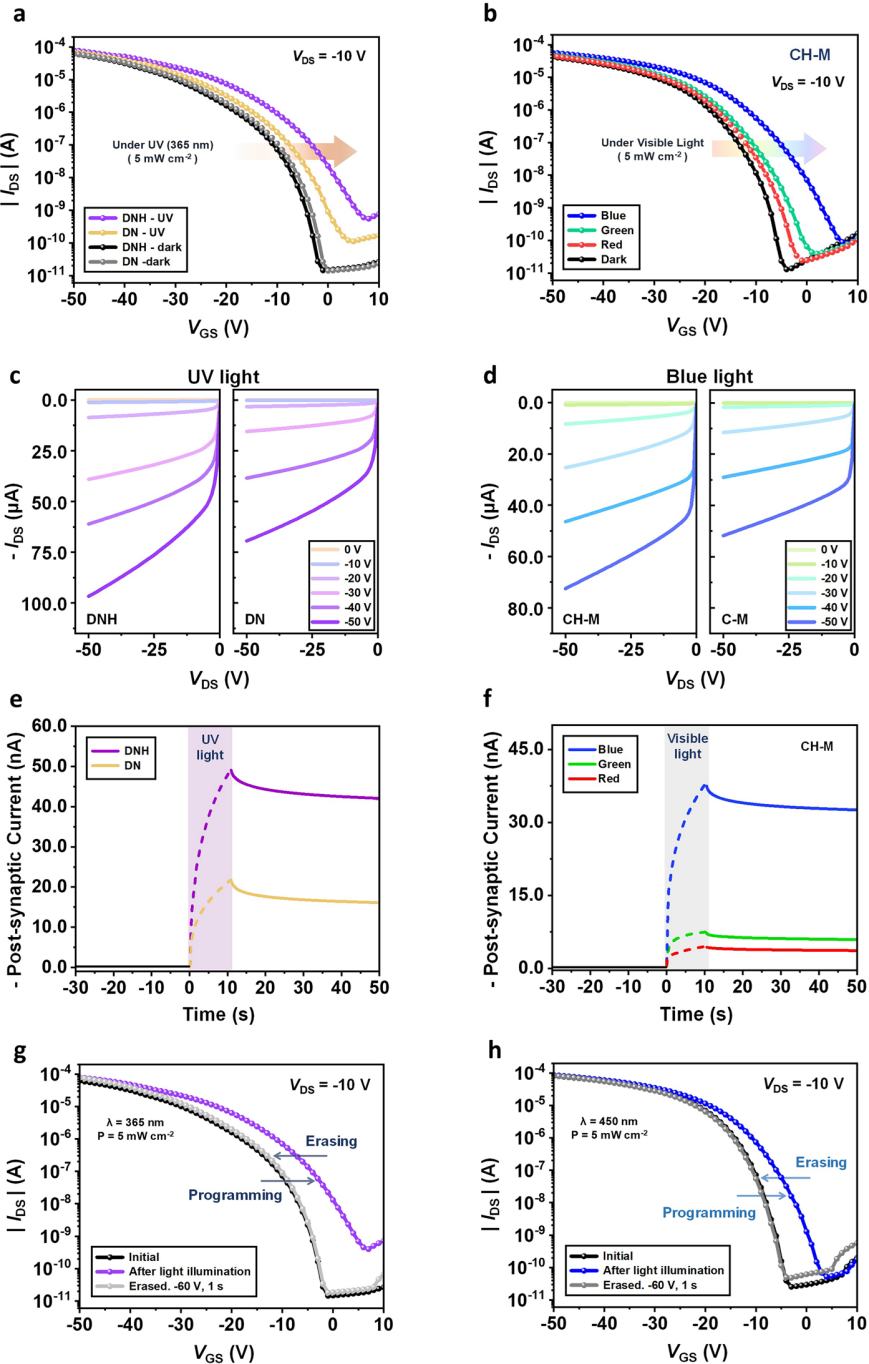
**a, b** Transfer curve variations of photonic synapse transistors under light illumination: (**a**) DNH and DN (365 nm UV light at 5 mW cm^−2^), (**b**) CH-M (450 nm blue, 525 nm green, and 630 nm red at 5 mW cm^−2^). **c, d** Output characteristics of photonic synapse transistors under light illumination (light power: 5 mW cm^−2^) with different *V*_G_ values: (**c**) DNH (left panel) and DN (right panel) at 365 nm UV light. (**d**) CH-M (left panel) and C-M (right panel) at 450 nm blue light. **e, f** Temporal changes in *I*_D_ of photonic synapse transistors under 10 s light illumination at *V*_D_ = −10 V: (**e**) DNH and DN at *V*_G_ = −1 V. (**f**) CH-M at *V*_G_ = −4 V. **g, h** Photo-programming and electrical erasing of photonic synapse transistors: (**g**) DNH (photo-programming pulse conditions: 365 nm UV light, 5 mW cm^−2^, and width of 10 s/electrical-programming pulse conditions: *V*_G_ = −60 V and width of 1 s). (**h**) CH-M (photo-programming pulse conditions: 450 nm blue light, 5 mW cm.^−2^, and width of 10 s/electrical-programming pulse conditions: *V*_G_ = −60 V and width of 1 s)

Figure 1d illustrates the energy-band diagrams of the components through the floating gate structure with an organic semiconductor body in the depletion mode at zero gate bias in thermal equilibrium. The energy levels of each layer are estimated by UPS and UV–Vis measurement data (Fig. S1). During photo-programming operation, numerous electron–hole pairs are generated within the light-responsive floating gate upon exposure to light and subsequently dissociated by the electric field present in the depletion mode. Under this electric field condition, the photogenerated holes can tunnel through the Al_2_O_3_ layer, while the photogenerated electrons become trapped in the conduction band of the floating layer, decreasing the *V*_th_ of the transistor (Fig. 2a, b, g, h). Conversely, during electrical-erasing operation, a negative voltage applied to the gate facilitates the removal of trapped electrons from the floating gate to the body, effectively restoring the *V*_th_ to its original value (Fig. 2g, h). Consequently, by employing light and voltage input, the *V*_th_ of the device can be decreased and increased, thereby altering the output current. Meanwhile, the trapped charges within the floating gate exhibit a semi-permanently long lifetime, leading to a non-volatile change in *V*_th_ even after the removal of the input signal. Therefore, this mechanism serves as the basis for the synaptic plasticity of the device, which will be discussed in detail later.

In the literature, multiple thin-film field-effect transistor (FET) works, which employed a photo-sensitive floating gate, have been reported. However, these devices often demonstrate a limited shift in the *V*_th_ when exposed to light [19–22]. This limitation can be attributed to the insufficient buildup of extra photoelectrons within the floating gate during light exposure, resulting in a minimal decrease in *V*_th_. In this work, we introduce an organic interlayer, capable of generating strong induced dipole moments upon excitation, as a photo-sensitive floating gate. This unique characteristic enables the accumulation of a higher number of extra photoelectrons within the floating gate when exposed to light, resulting in an expanded *V*_th_ shift window in response to the light signal. Moreover, this induced dipole moment effect provides the device with a remarkable color-discriminating capability, as it is dependent on the spectral absorbance and excited-state lifetime of the molecule. We will discuss this color functionality in detail later on.

In our recent investigation, we made a noteworthy discovery regarding the integration of an interlayer composed of an asymmetric molecule into devices that possess a high built-in electric field, such as photovoltaic cells and memristors. It has been observed that the asymmetric molecule, which exhibits a high intrinsic dipole moment, has the ability to generate induced dipole moment (= polarizability × electric field), align to the electric field, within the interlayer [32–34]. This is mainly from the electronic polarization in the molecules, fast enough due to the displacement of electron clouds. In particular, if the molecule has an enhanced intrinsic dipole moment at the excitation, the induced dipole moment effect within the interlayer is further enhanced at the light illumination. Importantly, this is effective with the molecules, which have extended lifetimes in their excited states. To obtain the molecule with extended lifetime at the excitation, we designed an asymmetric molecule to undergo an excited-state intramolecular proton transfer (ESIPT) process between enol (E*) and keto (K*) form at the excitation. The molecules that can have photo-tautomerization between E* and K* form at the excitation through intramolecular H-bond (e.g., –OH and –N=) are known to exhibit extended lifetime at the photo-excitation due to the stable K* form in the excited state (Fig. S2) [32–34].

In the context of the floating gate transistor device in this work, induced dipole moments are generated within the floating gate composed of asymmetric molecules (Fig. S3b, c) due to the inherent built-in potential (*V*_b_) (and built-in electric field (*E*_b_)), which in turn further enhance this potential within the device (*V*_b_ + *V*_d_) (and thus electric field (*E*_b_ + *E*_d_)). If the molecule possesses a high intrinsic dipole moment during excitation and exhibits an extended excitation lifetime (e.g., asymmetric ESIPT molecule), the induced dipole moment effect is further fortified within the floating gate under light exposure condition (Figs. 1i and S3d), and it can further maximize the electric field within the device (*E*_b_ + *E*_d_ at light). This maximized electric field promotes the effective dissociation of photogenerated electron–hole pairs, thereby increasing the number of trapped electrons within the floating gate. This, in turn, leads to a further reduction in *V*_th_ compared to the case without the induced dipole moment effect.

To validate the proposed concept, in which the variation window of the *V*_th_ in a photonic synapse transistor at the light irradiation can be further expanded through the floating gate comprising an ESIPT molecule, the device with 4-(di(naphthalen-2-yl)amino)-2-(1,4,5-triphenyl-1H-imidazol-2-yl)phenol (DNH: Fig. 1e) [33, 34] following ESIPT process is studied. Figure S4a shows that DNH, in its K* form, exhibits a significantly higher intrinsic dipole moment of 11.27 D during excitation compared to its E form, which possesses a dipole moment of 5.04 D in the ground state. Additionally, we prepared a device utilizing a non-ESIPT molecule, N-(naphthalen-2-yl)-N-(3-(1,4,5-triphenyl-1H-imidazol-2-yl)phenyl)naphthalen-2-amine (DN: Fig. 1f) [34], to emphasize the importance of extended lifetime during excitation through the ESIPT process for the effectiveness of the induced dipole moment effect. Furthermore, we extend this architecture to a device capable of superior RGB visible color discrimination by incorporating a visible light-absorbing ESIPT molecule, (E)-3-(4-(dimethylamino) phenyl)-1-(1-hydroxynaphthalen-2-yl)prop-2-en-1-one (CH-M: Fig. 1g) [34]. The device with its non-proton-transfer counterpart, (E)-3-(4-(dimethylamino)phenyl)-1-(naphthalen-2-yl)prop-2-en-1-one (C-M: Fig. 1h), is also investigated to confirm the importance of extended lifetime as a comparison [34]. The intrinsic dipole moments of CH-M and C-M are depicted in Fig. S4c, d. The color discrimination functionality of the device with CH-M will be discussed in the following section.

Prior to validating the suggested concept, we conducted experiments to confirm that the lifetimes of the molecules can be extended by designing them to undergo ESIPT process. Figure 1j, k depicts the time-resolved photoluminescence (TRPL) decay curves of DNH, DN, CH-M, and C-M films on a quartz glass substrate. The photoluminescence (PL) lifetimes of these organic thin films are obtained through bi-exponential fitting. The estimated PL lifetimes of DNH and CH-M, which undergo the ESIPT process, are found to be significantly longer compared to their non-proton-transfer counterparts, DN and C-M (Table S1). This suggests that the K* form in the ESIPT process contributes to the extension of the effective lifetimes upon photo-excitation.

Additionally, we perform UV–Vis spectroscopy measurement on DNH, DN, CH-M, and C-M in their thin-film states to confirm the absorbance of the molecules at the desired wavelength (Fig. 1c). For reference, the variation in UV–Vis spectra of DNH and CH-M films depending on prolonged light irradiation reveals that these molecules have a substantial stability against photodegradation (Fig. S5). We also examine the absorbance of C8-BTBT to ensure its visible transparency and minimal UV absorption. This is crucial to avoid any overlap with the absorbance profiles of DNH, DN, CH-M, and C-M taking into account that light is exposed from the C8-BTBT side. The energy-band diagrams of the components (before contact) [33, 34] are presented in Fig. S3a.

Before further investigating a detailed discussion of the color-discriminating functionality and photo-programming, we experimentally verify the strong excited-state dipole moment of the ESIPT-active molecules, DNH and CH-M. This was accomplished by characterizing the PL anisotropy (*r*) of the film, defined by the equation: *r* = (*I*_║_ − *I*_┴_)/(*I*_║_ + 2*GI*_┴_), where *I*_║_ (parallel) and *I*_┴_ (perpendicular) represent PL intensities with polarizers oriented in parallel and perpendicular geometries to the excitation light source, respectively. Both DNH and CH-M exhibit a significant difference between *I*_║_ and *I*_┴_ (within the wavelength range ± 5 nm, displaying maximum intensity), resulting in high film anisotropy (Fig. 1l, m). In contrast, conventional organic semiconductors lacking a strong excited-state dipole moment exhibit negligible anisotropy values, nearly approaching zero [32]. Moreover, we demonstrate that these excited-state dipole effects can align to the electric field by generating induced dipole moment within the film (e.g., electronic polarization resulting from the displacement of electron clouds, not orientation polarization, as mentioned). This alignment is critical for enhancing the electric field within the device, facilitating more efficient dissociation of photogenerated electron–hole pairs and consequently leading to a further reduction in threshold voltage (*V*_th_). The alignment of the electric field-oriented excited-state dipole moment is further assessed by observing the variation of PL anisotropy under an electric field. It is well established that the PL anisotropy of a sample reflects the dipole orientation within the sample [35, 36]. The chromophore molecules in the solid-state film exhibit limited rotation and negligible reabsorption, hence the relaxation of anisotropy provides insight into the arrangement of excited-state dipole moments. Figure 1n illustrates that the anisotropy values of both DNH and CH-M thin films gradually decrease with the increasing electric field, indicating a rapid arrangement of the excited-state dipole moments. For reference, the CH-M film exhibits a larger anisotropy variation than the DNH film.

### Color Discrimination Functionality

**Fig. 3:**
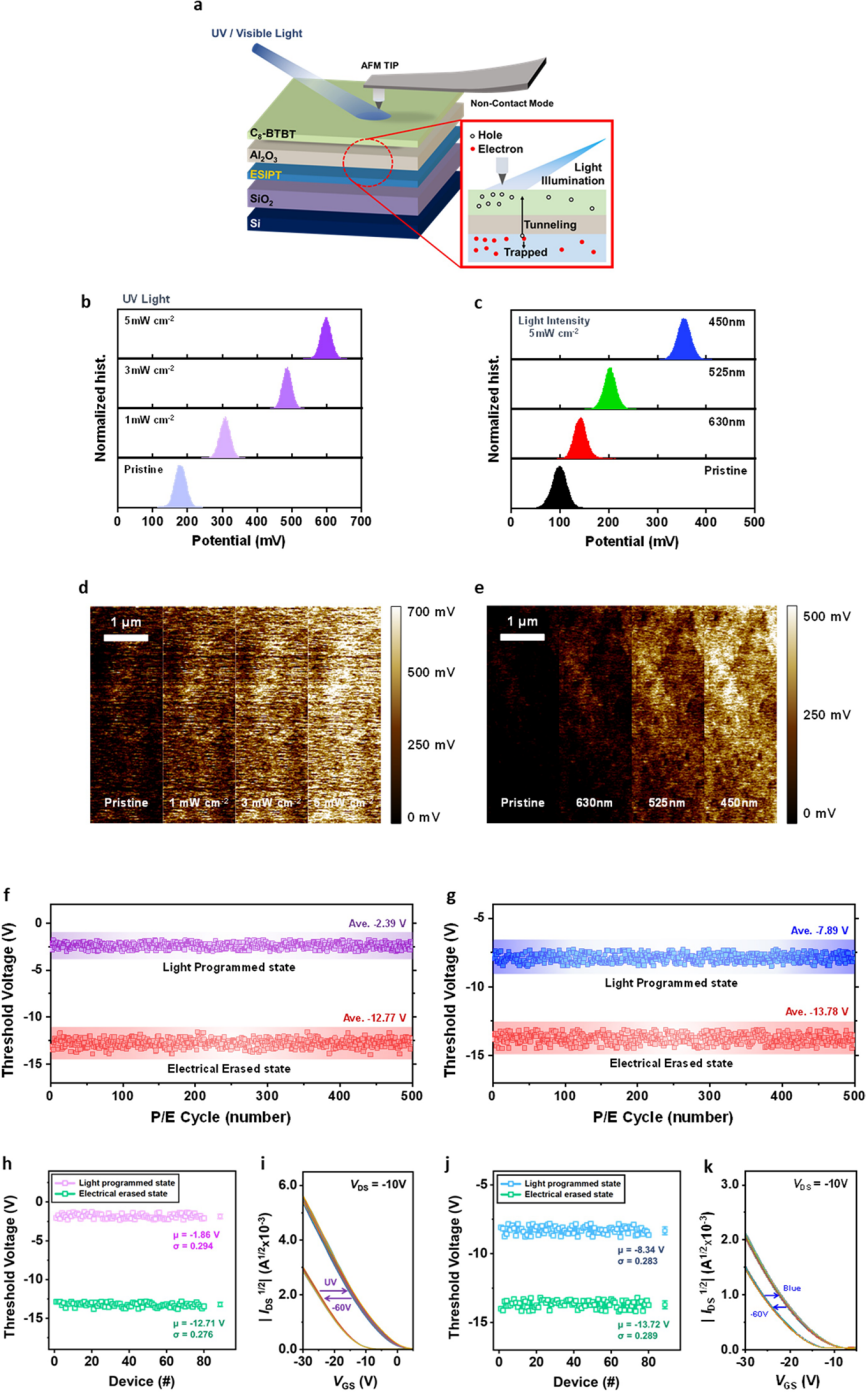
**a** Schematic illustration of KPFM measurement (Cr/Au conductive tip) on the C8-BTBT layer in device architecture. The scanning area and scan rate are 5 × 5 μm^2^ and 0.3 Hz. For surface potential measurement, the hybrid film-tip distance is 75 nm for all measurement conditions (resolution: 256 pixels × 256 pixels). **b-e** Measured surface potentials of C8-BTBT layers on Si/SiO_2_/ESIPT-organic semiconductors/Al_2_O_3_: (**b, c**) Distributions and (**d, e**) 2D maps. (**b, d**) UV light illumination (365 nm) on DNH-integrated sample at different light intensities, and (**c, e**) RGB light illumination on CH-M integrated sample at fixed light intensity. **f, g** Cycle-to-cycle variation (CCV) of (**f**) DNH- and (**g**) CH-M-integrated photonic synapse transistors. *V*_th_ values in photo-programmed states and electrically erased states during 500 continuous cycles in ambient conditions. **h-k** Device-to-device variation (DDV) of (**h, i**) DNH- and (**j, k**) CH-M-integrated photonic synapse transistors. (**h, j**) Statistics of *V*_th_ values and (**i, k**) transfer curves in photo-programmed state and electric erased state from 80 devices. Photo-programming pulse conditions of DNH-case: 365 nm UV light, 5 mW cm^−2^, and width of 10 s/electrical-programming pulse conditions of DNH-case: *V*_G_ = −60 V and width of 1 s. Photo-programming pulse conditions of CH-M-case: 450 nm blue light, 5 mW cm^−2^, and width of 10 s/electrical-programming pulse conditions of CH-M-case: *V*_G_ = −60 V and width of 1 s

Using in situ Kelvin probe force microscopy (KPFM), we present evidence indicating the shift in *V*_th_ upon light illumination primarily arises from potential variations between the Si gate and the C8-BTBT semiconductor body (Fig. 3a). These variations are attributed to the presence of trapped photoelectrons within the floating gate. Figure 3b, d presents the measured contact potential difference (CPD) of the C8-BTBT film (on Si/SiO_2_/DNH/Al_2_O_3_), demonstrating a significant increase under UV light. The average surface potential gradually rises from 188.31 to 309.72, 489.72, and 607.01 mV as the light intensity increases from 0 to 1, 3, and 5 mW cm^−2^, respectively (Fig. 3b, d). This observation suggests that higher light intensity results in a more pronounced potential change due to an increased amount of trapped charge.

This behavior extends to a color-discriminating characteristic. Under light irradiation with different wavelengths, the number of trapped charges varies, influenced by three factors. Firstly, the spectral absorbance gradient of the molecule thin film leads to different numbers of generated photoelectrons. Secondly, the ESIPT molecule thin film, with its spectral absorbance gradient, provides varying strengths of induced dipole moment depending on its absorbance. This is because higher absorbance corresponds to a greater number of excited molecules with higher dipole moments upon excitation. In addition to these spectral absorbance gradient-related properties, the wavelength-dependent excited-state lifetime of the ESIPT molecule, which is directly linked to the efficiency of the induced dipole moment, is another crucial factor. The excited-state lifetime of the ESIPT molecule tends to increase as the wavelength of the incident light decreases. This occurs because higher-energy photons at shorter wavelengths possess sufficient energy to facilitate efficient and rapid proton transfer processes from E* to K*, thereby stabilizing the excited state and extending its lifetime [37–39]. Figure S6 validates that the PL lifetime of CH-M, when excited at shorter wavelengths, is much longer than that observed when excited at longer wavelengths. Therefore, by introducing the ESIPT-active CH-M layer with varying levels of absorbance and excited-state lifetime in the visible range depending on the wavelength (Figs. 1c and S5b: highest in blue light and lowest in red light), we can expect different degrees of potential variation under light of different wavelengths. Further exploration of the wavelength-dependent functionality of the device will be discussed in subsequent sections.

Figure 3c, e illustrates the exposure of the C8-BTBT film (on Si/SiO_2_/CH-M/Al_2_O_3_) to visible light with a fixed intensity of 5 mW cm^−2^ and wavelengths ranging from 630 to 450 nm. The CPD of the C8-BTBT film steadily increases from 99.27 to 138.66, 211.19, and 368.81 mV as the wavelength of light illumination transitions from dark to 630, 525, and 450 nm, respectively. This wavelength-dependent potential variation serves as a foundation for the color discrimination capability of the photonic transistor. For reference, the spectra of UV and RGB light employed in this work are depicted in Fig. S7.

### Photo-Programming

Figure 2a illustrates the transfer characteristics of the transistors incorporating the DNH and DN as floating gate layers under UV light with an intensity of 5 mW cm^−2^. Both transfer curves exhibit a shift toward the positive direction, indicating a decrease in *V*_th_ and thus an increase in *I*_D_ at the same gate voltage (*V*_GS_). Notably, the device incorporating the ESIPT-active DNH exhibits a further reduction in *V*_th_ compared to the non-ESIPT DN device, as intended. This result highlights the significance of the long-lasting photo-enhanced dipoles generated through the ESIPT process—leading to strong induced dipole effect—which are critical for the enhanced photo-functionality of our photonic transistor. Likewise, the output characteristics of both transistors demonstrate an increase in *I*_D_ under UV light illumination compared to the dark condition (Fig. S8a, c). Furthermore, the device incorporating DNH exhibits significantly higher *I*_D_ than the one with DN under the same UV power condition (Fig. 2c).

The temporal changes in *I*_D_, measured with and without UV light at an intensity of 5 mW cm^−2^, are represented in Fig. 2e. Upon UV light illumination, the current experiences a sudden and pronounced increase. Notably, the device with an ESIPT floating gate exhibits a higher current increase compared to the device with a non-ESIPT floating gate, which is consistent with the previously observed *V*_th_ variation. Interestingly, even after the light sources are turned off, the current gradually decreases and does not return to its initial level. This indicates a permanent change in the conductance of the device, which comes from the trapped photoelectron within the floating gate.

The light-responsive capabilities of the photonic transistor can be expanded into the visible region by introducing a chalcone derivative CH-M, which undergoes the ESIPT process upon visible light illumination, and C-M, which is a non-ESIPT molecule derived from CH-M for comparison purposes, as the floating gate. As mentioned earlier, the higher absorbance of CH-M in the blue color region leads to the generation of a larger number of photoelectrons compared to other colors. CH-M exhibits the strongest (weakest) induced dipole effect under blue light (red light) due to its higher absorbance and longer PL lifetime at shorter wavelengths. These unique characteristics give rise to varying levels of *V*_th_ shift depending on the incident color, enabling RGB color discrimination in the CH-M-integrated photonic transistor.

Consequently, the transfer characteristics of the transistor incorporating the CH-M as floating gate layers exhibit distinct shifts toward the positive direction, depending on the wavelength of the visible light, even when subjected to the same power condition (5 mW cm^−2^) (Fig. 2b). This feature allows the device to possess color discrimination ability. Therefore, the temporal changes in *I*_D_ exhibit diverse levels of current increase when subjected to different colored light, while the corresponding non-volatile conductance changes also vary according to the wavelength (Fig. 2f). Consequently, the light illumination can be effectively employed as a photo-programming process, and this non-volatile conductance variation serves as the fundamental basis for the synaptic property. The monochromatic light sources with peak wavelengths of 450 nm (blue), 525 nm (green), and 630 nm (red) (Fig. S7) were used at an equal intensity of 5 mW cm^−2^. The output characteristics of the transistor with CH-M are depicted in Figs. 2d and S8b, d. For reference, the transfer characteristics of the C-M-integrated device along with its temporal changes in *I*_D_ under three different light illuminations (RGB) are presented in Fig. S9. Similar to the performances of the DN-integrated device, the *V*_th_ shift and *I*_D_ increase of the C-M-integrated device upon light illumination are significantly lower compared to those of the CH-M-integrated device.

The photo-programmable and electrical-erasable transfer characteristics of DNH- and CH-M-based flash memory devices are presented in Fig. 2g, h. UV-light pulse with a wavelength of 365 nm, intensity of 5 mW cm^−2^, and width of 10 s was employed as programming operation while the electrical gate voltage (*V*_G_) pulse with amplitude of -60 V and width of 1 s was applied as erasing operation. As for the transistor with the CH-M interlayer, photo-programming under blue light (450 nm, 5 mW cm^−2^, and width of 10 s) in conjunction with its corresponding electrical programming (−60 V and 1 s) is represented as a representative. After the light programming operation, the transfer curves of each DNH- and CH-M-integrated transistor undergo a significant shift toward the positive direction. This shift indicates a non-volatile decrease in *V*_th_, attributed to the trapping of electrons in DNH and CH-M. Following the electrical erasing operation, the *V*_th_ can be switched back to its initial state, signifying the release of the trapped electrons. For comparison, a C8-BTBT transistor without the floating gate layer was fabricated and exposed to light pulses (UV and blue light). As shown in Fig. S10, the device exhibited negligible *V*_th_ shift compared to the DNH- and CH-M-integrated devices. The slight decrease in *V*_*t*h_ upon light irradiation is likely attributed to the photoconductivity effect [32, 34].

The repetitive cycles of photo-programming and electrical erasing in flash memory devices integrated with ESIPT-active organic thin films, such as DNH and CH-M, under ambient condition are depicted in Fig. 3f, g. The memory window, defined as the difference between *V*_th_ in the programmed (365 and 450 nm light pulse for DNH and CH-M with intensity of 5 mW cm^−2^ and width of 10 s) and erased state (*V*_G_ pulse with amplitude of −60 V and width of 1 s), is maintained without significant degradation after 500 continuous cycles, indicating excellent reproducibility and endurance of the devices with low cycle-to-cycle variation (CCV). To access thermal stability, we conducted photo-programming and electrical erasing at elevated temperatures ranging from room temperature (25 °C) to 130 °C, showing normal behaviors with slight fluctuations in the transfer curves of DNH and CH-M integrated transistor (Fig. S11). Furthermore, device-to-device variation (DDV) is also investigated through the analysis of the transfer curves and memory windows obtained from 80 devices each for DNH- and CH-M-integrated flash memory devices (Fig. 3h, i and j, k, respectively). The narrow distributions indicate high reproducibility across multiple devices.

### Photo-Synaptic Properties

As previously mentioned, the trapped photoelectrons within the floating gate confer synaptic characteristics to our device. In this section, we delve deeper into the detailed synaptic properties of the device, paving the way for its potential extension in the artificial visual system, which will be discussed in the last section.

**Fig. 4:**
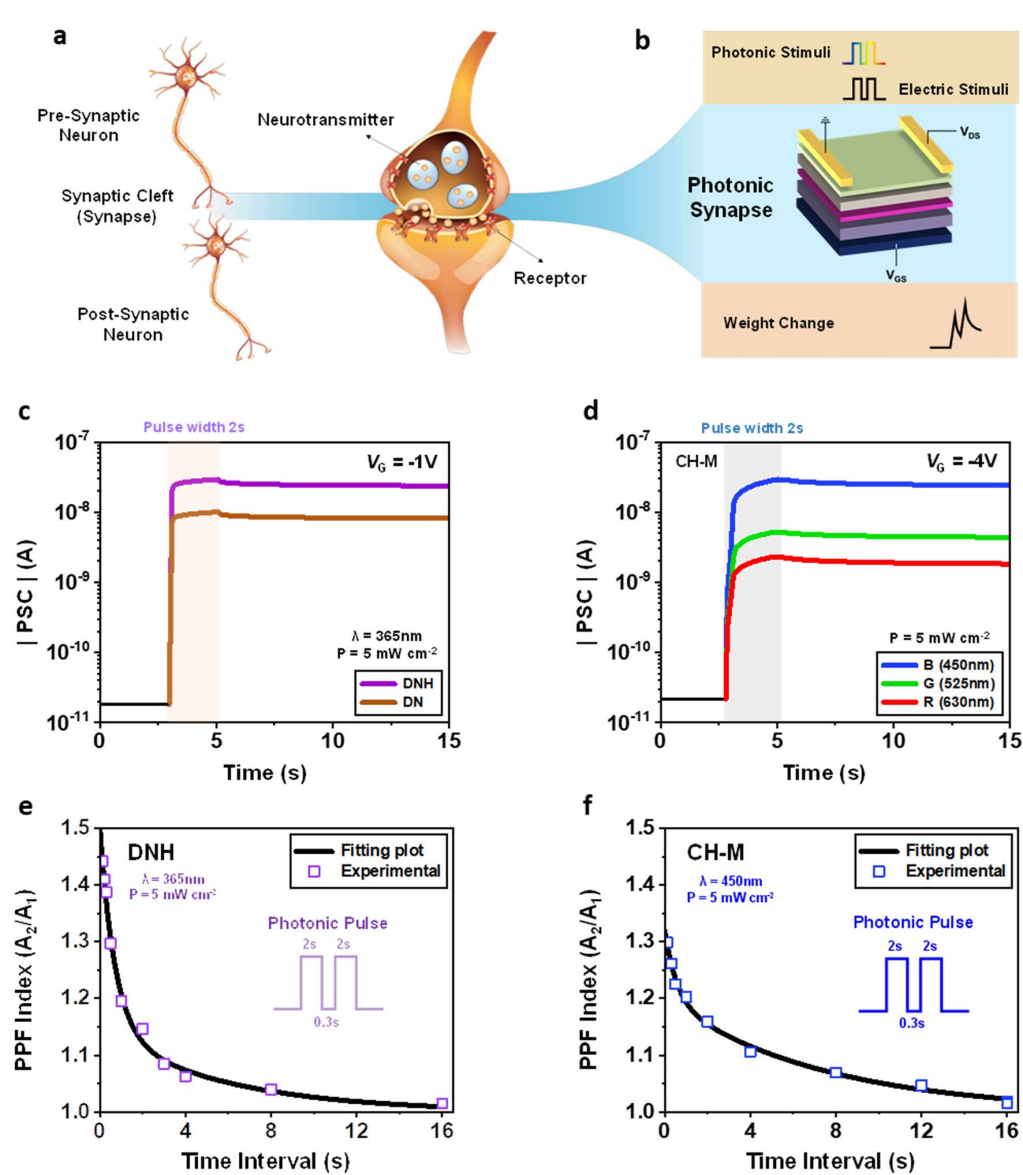
**a, b** Schematic illustrations of functional and architectural comparisons of (**a**) biological synapse with (**b**) photonic synapse in this work. **c, d** EPSC behaviors of photonic synapses at a readout voltage *V*_DS_ = −10 V: (**c**) DNH and DN (365 nm UV light at 5 mW cm^−2^ with 2 s pulse width) at *V*_G_ = −1 V. (**d**) CH-M (450 nm blue, 525 nm green, and 630 nm red at 5 mW cm^−2^ with 2 s pulse width) at *V*_G_ = −4 V. **e, f** PPF index (*A*_2_/*A*_1_) as a function of interval between two consecutive light pulses (0.1 s ≤ Δ*t* ≤ 16 s) at readout voltage *V*_DS_ = −10 V: (**e**) DNH (365 nm UV light at 5 mW cm^−2^ with 2 s pulse width) at *V*_G_ = −1 V. (**f**) CH-M (450 nm blue light at 5 mW cm^−2^ with 2 s pulse width) at *V*_G_ = −4 V

The biological synapse serves as a crucial link between the neurons, acting as a functional unit within the human nervous system responsible for complex tasks such as learning, memory, and processing [40, 41]. As depicted in Fig. 4a, when an action potential reaches the pre-synapse, neurotransmitters are released into the synaptic cleft, leading to potential variations in the post-synapse [42, 43]. The excitatory or inhibitory posts-synaptic current (EPSC or IPSC) depends on the strength of the synapse (e.g., synaptic weight), typically determined by the number of receptors involved [44]. In our photonic transistor (Fig. 4b), the conductance of the device, representing the synaptic weight, can be modulated by employing light pulses on the photo-responsive floating gate and electrical pulses on the Si gate.

Figure 4c, d illustrates the EPSC behaviors of DNH-, DN-, and CH-M integrated photonic transistors. These behaviors are evoked by a single optical pulse spike as a pre-synaptic signal (DNH, DN: peak wavelength = 365 nm, CH-M: multiple peak wavelengths = 450, 525, and 630 nm at intensity of 5 mW cm^−2^ and width of 2 s). The EPSCs are recorded at a constant reading voltage of *V*_D_ = −10 V and *V*_G_ = −1 V for DNH and DN, and *V*_D_ = −10 V and *V*_G_ = −4 V for CH-M. The post-synaptic current (PSC) of the devices exhibits a rapid increase upon the arrival of the input light pulse and then gradually decays, settling at a certain current level even after the pulse is turned off. Figure S12 illustrates that both the increase of PSC during the light pulse and the persistent conductance change following the pulse are directly correlated with the intensity of light pulse. The non-volatile conductance change become the origin of the long-term plasticity in the photonic synapse. Under specific light illuminations for the ESIPT-active cases (365 nm for DNH and 450 nm for CH-M, with 5 mW cm^−2^ of intensity and 20 ms of pulse width) at *V*_D_ = −0.05 V, the estimated energy consumption values for reading are 15.05 and 14.37 fJ, respectively, per synaptic event (Fig. S13), which are compatible with that of biological synapse [45].

Figure 4c confirms that the ∆EPSC of the transistor with ESIPT-active DNH is significantly higher than that of the non-ESIPT DN under UV light pulse, which corresponds well with the *V*_th_ variation observed during light illumination, as previously described (Fig. 2). Furthermore, the CH-M-integrated transistor demonstrates varying levels of ∆EPSC in response to three distinct monochromatic visible light pulses (RGB) of equal intensity and width (Fig. 4d). This suggests the possibility of color-distinguishable photo-synaptic characteristics. For reference, the EPSC behavior of C-M-integrated device, using the same input signals as CH-M, is considerably weaker (Fig. S14), similar to the DN-integrated device.

As a fundamental parameter for evaluating the short-term plasticity of synaptic devices, the paired-pulse facilitation (PPF) behavior, which is induced by two consecutive light pulses, is investigated by varying time intervals between the paired pulses from 0.1 to 16 s. Figures 4e, f and S15 illustrate the PPF indices of DNH- and CH-M-integrated devices, which are defined as the ratio of the second post-synaptic current amplitude (*A*_2_) to that of the first post-synaptic current amplitude (*A*_1_) (Fig. S16) [46]. Notably, these indices steadily increase as the pulse interval decreases. These experimentally estimated PPF characteristics can be fitted by the following double-exponential function:

1 $$\text{PPF index}={C}_{1}\cdot \text{exp}\left(-\frac{\Delta t}{{\tau }_{1}}\right)+{C}_{2}\cdot \text{exp}\left(-\frac{\Delta t}{{\tau }_{2}}\right)+1$$

Here, *τ*_1_ and *τ*_2_ represent the fast and slow phase relaxation time, respectively, while *C*_1_ and *C*_2_ denote the initial facilitation of those phases [47]. *τ*_1_ and *τ*_2_ values of the transistor with various organic semiconductors are summarized in Table S2. It is worth noting that the PPF behavior, along with the EPSC behavior, can be accompanied by non-volatile conductance adjustment of the photonic transistor. This feature provides a basis for converting short-term memory to long-term memory, resembling the learning behaviors observed in the brain.

**Fig. 5:**
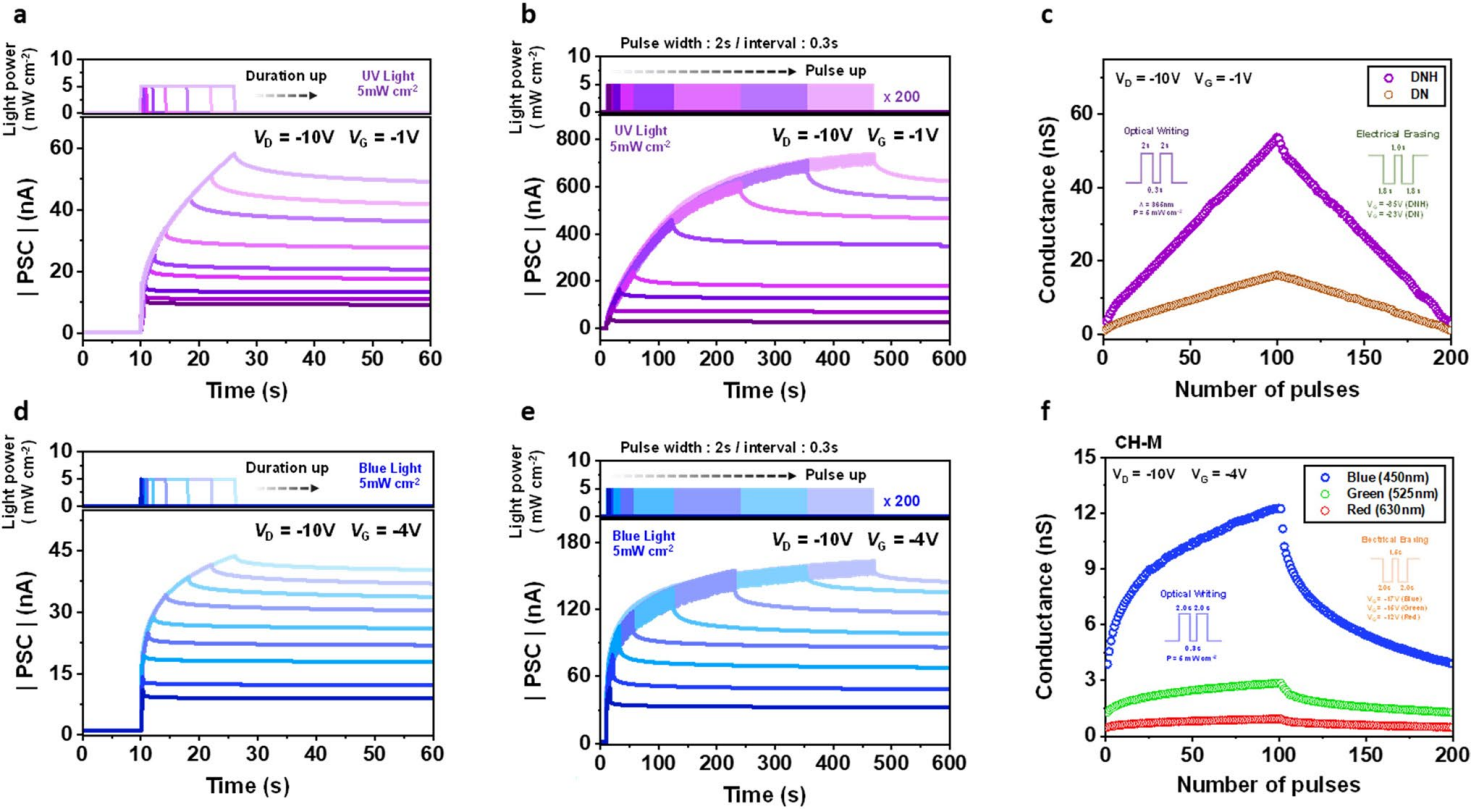
**a, b, d, e** PSC variations of photonic synapse transistors depending on various light pulse conditions. Pulse width: (**a**) DNH and (**d**) CH-M. Light pulse conditions are described in top figures. Pulse number: (**b**) DNH and (**e**) CH-M. Light pulse conditions are depicted in top figures. **c, f** LTP/LTD characteristics of photonic synapse transistors: (**c**) DNH and DN, and (**f**) CH-M. Light and voltage pulse conditions for LTP and LTD are depicted in inset figures

Figure 5a, d represents the variation in PSC of DNH- and CH-M-integrated devices in response to varying light pulse widths. As the duration of the light pulse increases from 0.1 to 16 s, the corresponding PSC values gradually change, reaching 10.39–58.22 nA for DNH and 9.73–43.81 nA for CH-M. Furthermore, the PSC responses of both photonic synapses exhibit effective enhancement with an increasing number of consecutive light pulses. Specifically, in the case of DNH with UV light (365 nm, 5 mW cm^−2^, duration: 2 s, and interval: 0.3 s) and CH-M with blue light (450 nm, 5 mW cm^−2^, duration: 2 s, and interval: 0.3 s), the PSCs are significantly amplified during repetitive stimulation with up to 200 light pulses, reaching 735.21 nA (Fig. 5b) and 163.15 nA (Fig. 5e), respectively. These results confirm that light can effectively function as an input signal, providing an output current from the device.

Memory and learning behaviors are commonly attributed to the interplay between short-term plasticity (STP) and long-term plasticity (LTP). Through the influence of preceding neural activities, facilitated by repeated rehearsal events [48], short-term memory undergoes a conversion process, transitioning into long-term memory. Our artificial photonic synapses, utilizing a light-responsive ESIPT-active interlayer, successfully implement the fundamental synaptic behavior of STP-LTP transformation. Figure 5c illustrates long-term potentiation/long-term depression (LTP/LTD) curves of DNH- and DN- integrated devices, representing their conductance modification through a series of pulses. The experiment consists of 100 optical potentiation steps (365 nm light pulses: 5 mW cm^−2^ amplitude, 2 s width, and 0.3 s interval) followed by 100 electrical depression steps [*V*_G_ pulses: −35 V (DNH)/−23 V (DN) amplitudes, 1.8 s width, and 1 s interval]. It is observed that the DNH-integrated device exhibits a more efficient response to the light pulse signal compared to the DN-integrated device, highlighting the significance of the long-lasting strong photo-enhanced dipole moment once again.

In addition, the CH-M-integrated photonic synapse exhibits distinct responses to each monochromatic visible light pulses, demonstrating separate LTP/LTD curves for different colors and highlighting the color-dependent distinguishable capacity of the photonic transistor (Fig. 5f). During the optical potentiation steps, a series of 100 monochromatic light pulses (blue: 450 nm, green: 525 nm, red: 630 nm, 5 mW cm^−2^, 2 s width, and 0.3 s interval) are applied to the device to increase its conductance. Conversely, in the electrical depression steps, 100 optimized negative *V*_G_ pulses (blue: −17 V, green: −15 V, red: −12 V, 2 s width, and 1.5 s interval) are employed to decrease the conductance. For evaluating the characteristics of the measured LTP/LTD conductance curves, the nonlinearity coefficient (1/*A*_P_ and 1/*A*_D_) values of the LTP/LTD curves for all cases (DNH, DN, and CH-M under UV and visible light illumination) are estimated in Fig. S17. The calculation details regarding the nonlinearity are described in Note S2. For better understanding, Fig. S18 presents theoretically estimated LTP/LTD curves with nonlinearity values (1/*A*) ranging from 0 to 10.

### Color-Discriminating Photo-Synaptic Properties

**Fig. 6:**
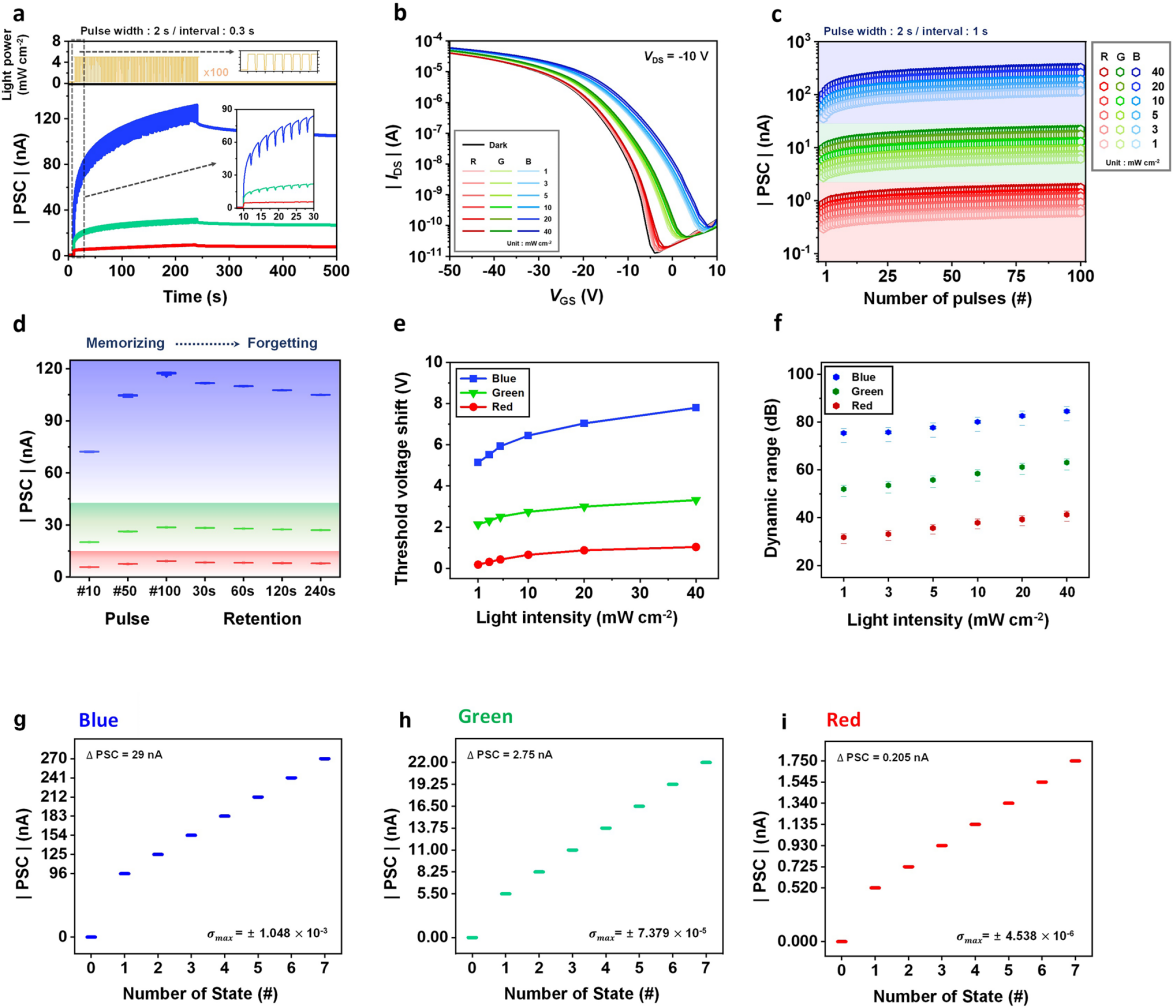
**a** PSC variations of CH-M-integrated photonic synapse under 100 consecutive pulses of monochromatic visible light (R: 630 nm, G: 525 nm, and B: 450 nm) at the same light pulse conditions (5 mW cm^−2^ intensity, 2 s width, and 0.3 s interval). Light pulse conditions are depicted in top figure. *V*_GS_ and *V*_DS_ are −4 V and −10 V. **b** Transfer curves of the CH-M-integrated photonic synapse after RGB single light pulse (2 s width) with various light intensities from 1 to 40 mW cm^−2^. **c** PSC distribution of CH-M integrated photonic synapse as a function of the number of RGB light pulses (2 s width) under various light intensities from 1 to 40 mW cm^−2^. **d** PSC distribution obtained from (**a**) depending on the number of pulses and retention time. PSCs are recorded immediately after 10th, 50th, 100th pulses, and 30, 60, 120, and 240 s after the last pulse. The average and standard deviation values are from 50 devices. **e** *V*_th_ shift estimated from the transfer curves in (**b**). **f** Dynamic range of CH-M-integrated photonic synapse calculated from the PSC distribution in (**c**). Circles are average values, and bars represent maximum and minimum values. **g-i** RGB-mixed color discrimination of CH-M-integrated photonic synapse (C8-BTBT channel) with 3-bit resolution: (B, G, R) = (0–7, 0–7, 0–7). PSC values of each state are obtained from the PSC values at 100th pules [0, 1, 3, 5, 10, 20, 30 and 40 mW cm^−2^ in (**c**)]. Standard deviation values depicted in each figure are estimated from 30 individual devices under the same conditions in (**c**). (**g**) blue, (**h**) green, and (**i**) red

Figure 6a demonstrates the variation in PSC and conductance change of the CH-M-integrated transistor during 100 consecutive monochromatic light pulses (blue: 450 nm, green: 525 nm, red: 630 nm, 5 mW cm^−2^). The figure illustrates that RGB color discrimination becomes more distinct with an increasing number of light pulses. Furthermore, the presence of stable retention properties, as evidenced by the maintenance of gaps (Fig. S19), is observed even after pulse, which can be attributed to the non-volatile conductance change. The output distribution of PSC (and the corresponding conductance) is clearly separated for the three primary colors and remains preserved even after the light pulses (Fig. 6d).

In order to validate the color discrimination ability of the photonic synapse in different external environments, we conducted an investigation on the color distinction of the CH-M-based device under various intensity ranges, specifically ranging from 1 to 40 mW cm^−2^ (Fig. 6b, c, e, f), which are applicable to human visual systems and considered safe for human eye [49, 50]. This intensity range is much higher than what has been reported in the literature [9]. We posit that the strong induced dipole moment effect during excitation, which enhances the trapped photo-carrier effect, expands the *V*_th_ variation window, enabling efficient color discrimination even within a wide intensity range.

Under monochromatic light pulses of red (630 nm), green (525 nm), and blue (450 nm) within the intensity range from 1 to 40 mW cm^−2^, the *V*_th_ shifts are distinctly separated. Specifically, for red light pulse, the *V*_th_ shifts range from 0.18 to 1.04 V; for green light pulse, the range is from 2.14 to 3.32 V; and for blue light pulse, the range extends from 5.13 to 7.81 V. These results are illustrated in Fig. 6b, e. Furthermore, the contrasts between the discriminated colors within the mentioned intensity range become more distinct after a 100 consecutive pulse train (Fig. 6c). This clear color distinction can be quantified by the dynamic range, which represents the ratio of the measurable PSC signal (PSC values under each RGB colored light with various intensities and pulse conditions) to the smallest measurable signal [PSC_min_: PSC value under the minimum light condition (red light with an intensity of 1 mW cm^−2^ and 1^st^ pulse)] expressed in decibels (dB). The dynamic range for current, as shown in Fig. 6f, can be calculated using Eq. (2):

2 $$\text{Dynamic range }\left(\text{dB}\right)=20 \text{log}(PSC /{PSC}_{\text{min}})$$

The dynamic range values for RGB colors are as follows: for red, the range is from 27.4 to 42.8 dB; for green, it is from 46.7 to 64.8 dB; and for blue, the range spans from 67.4 to 86.6 dB. These results further confirm the significant discrimination capability of the CH-M-integrated device about RGB colors across various available intensity range, due to its non-overlapping output current responses. For reference, our photonic synapse also maintains color-discrimination capabilities even under the conditions with the light intensity below 1 mW cm^−2^. The PSC distributions of CH-M-integrated photonic synapse subjected to consecutive pulses of RGB light with intensities ranging from 0.05 to 1 mW cm^−2^ are represented in Fig. S20.

Under RGB-mixed light illumination, our CH-M-integrated transistor demonstrates the capability to distinguish mixed colors into individual RGB components with 3-bit resolution [e.g., (R, G, B) = (0–7, 0–7, 0–7)]. Figure 6g–i and Table S3 present an exemplary 3-bit resolution mixed RGB color-discrimination. For the photonic synapse to possess the mixed color discrimination capability, PSC and conductance changes must be distinct for each RGB primary colors, as described, and the difference in PSC values between each state of B color should significantly exceed the highest PSC value among the states of G and R colors. Similarly, the difference in PSC values between each state of G color should surpass the highest PSC value among states in R color. Additionally, for valid color-discrimination, the deviation of PSC values for B color at each state must be considerably smaller than the gap between the states for G and R colors, and the deviation of PSC values for G color at each state should also be smaller than the gap between the states of R color. Further details are expounded in Note S3.

**Fig. 7:**
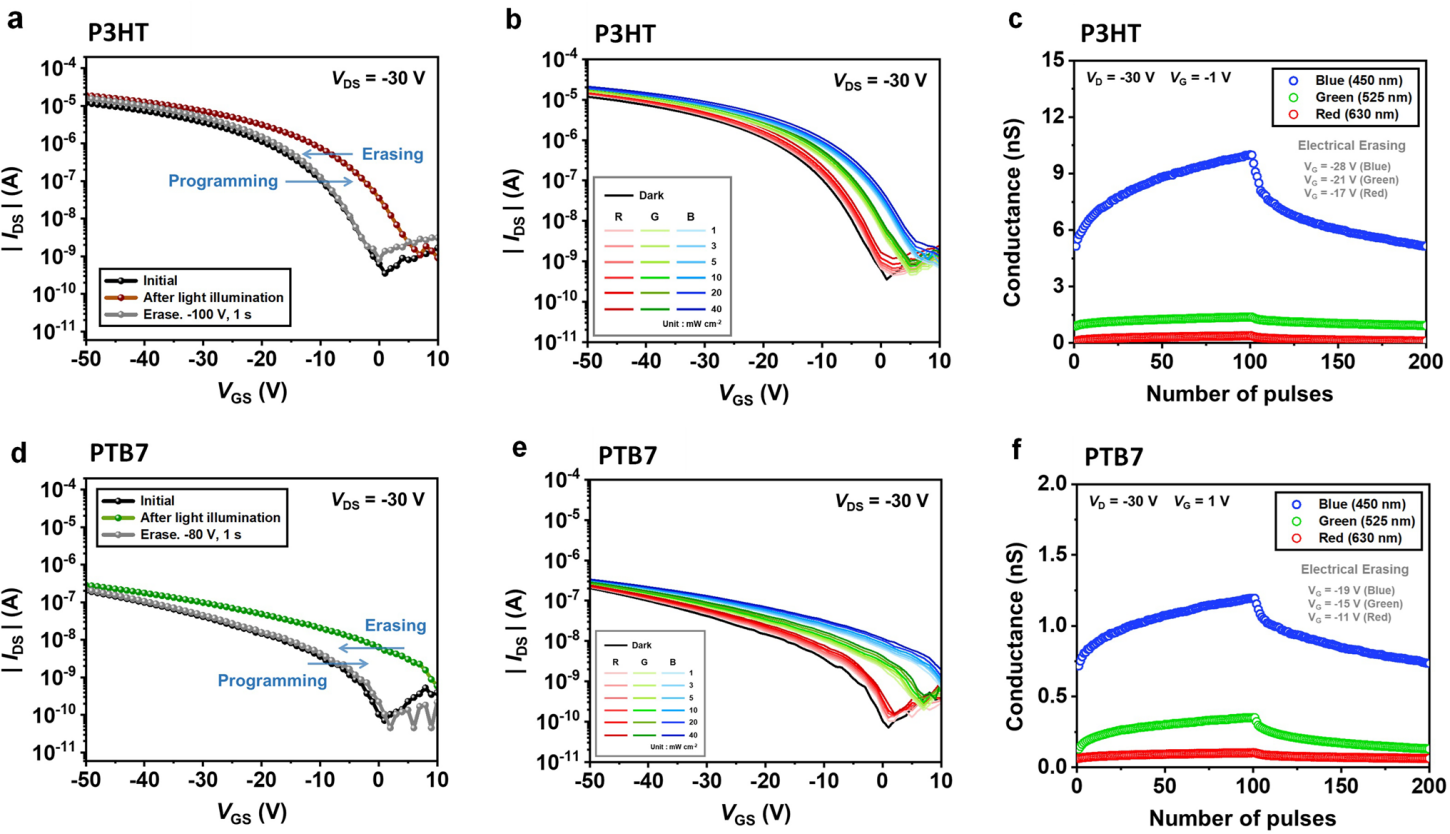
**a, d** Photo-programming and electrical erasing of photonic synapse transistors depending on various semiconductor channel medium (photo-programming pulse condition: 450 nm blue light, 5 mW cm^−2^, and width of 10 s): (**a**) P3HT (electrical-programming pulse condition: *V*_G_ = −100 V and width of 1 s at *V*_D_ = −30 V), (**d**) PTB7 (electrical-programming pulse condition: *V*_G_ = −80 V and width of 1 s at *V*_D_ = −30 V. **b, e** Transfer curves of photonic synapse depending on various semiconductor channel medium after RGB single light pulse (2 s width) with various light intensities from 1 to 40 mW cm^−2^: (**b**) P3HT at *V*_D_ = −30 V, (**e**) PTB7 at *V*_D_ = −30 V. **c, f** LTP/LTD characteristics of photonic synapse transistors depending on various semiconductor channel medium: (**c**) P3HT at *V*_D_ = −30 V, *V*_G_ = −1 V. (**f**) PTB7 at *V*_D_ = −30 V, *V*_G_ = 1 V. Light pulse conditions for LTP are same as depicted in Fig. 5f inset figures. Voltage pulse conditions for LTD are same as pulse width 2 s and pulse interval 1.5 s under the different pulse amplitudes: P3HT (pulse amplitudes: blue = −28 V, green = -21 V, and red = −17 V), and PTB7 (pulse amplitudes: blue = −19 V, green = −15 V, and red = −11 V)

Meanwhile, we fabricated CH-M-integrated photonic synapses employing diverse channel materials and examined their photo-functionality to establish the universality of our approach. Two widely utilized organic semiconductors, namely P3HT and PTB7, were introduced as alternative channel materials. The results, depicted in Fig. 7a–f, demonstrate their capability to successfully execute photo-functionality tasks, including photo-programming, electrical erasing, color discrimination under various light intensities ranging from 1 to 40 mW cm^−2^, and LTP/LTD behaviors under RGB colored lights. For reference, the optical properties of P3HT and PTB7, including their UV–visible absorption spectra and optical bandgap, are represented in Fig. S21.

**Fig. 8:**
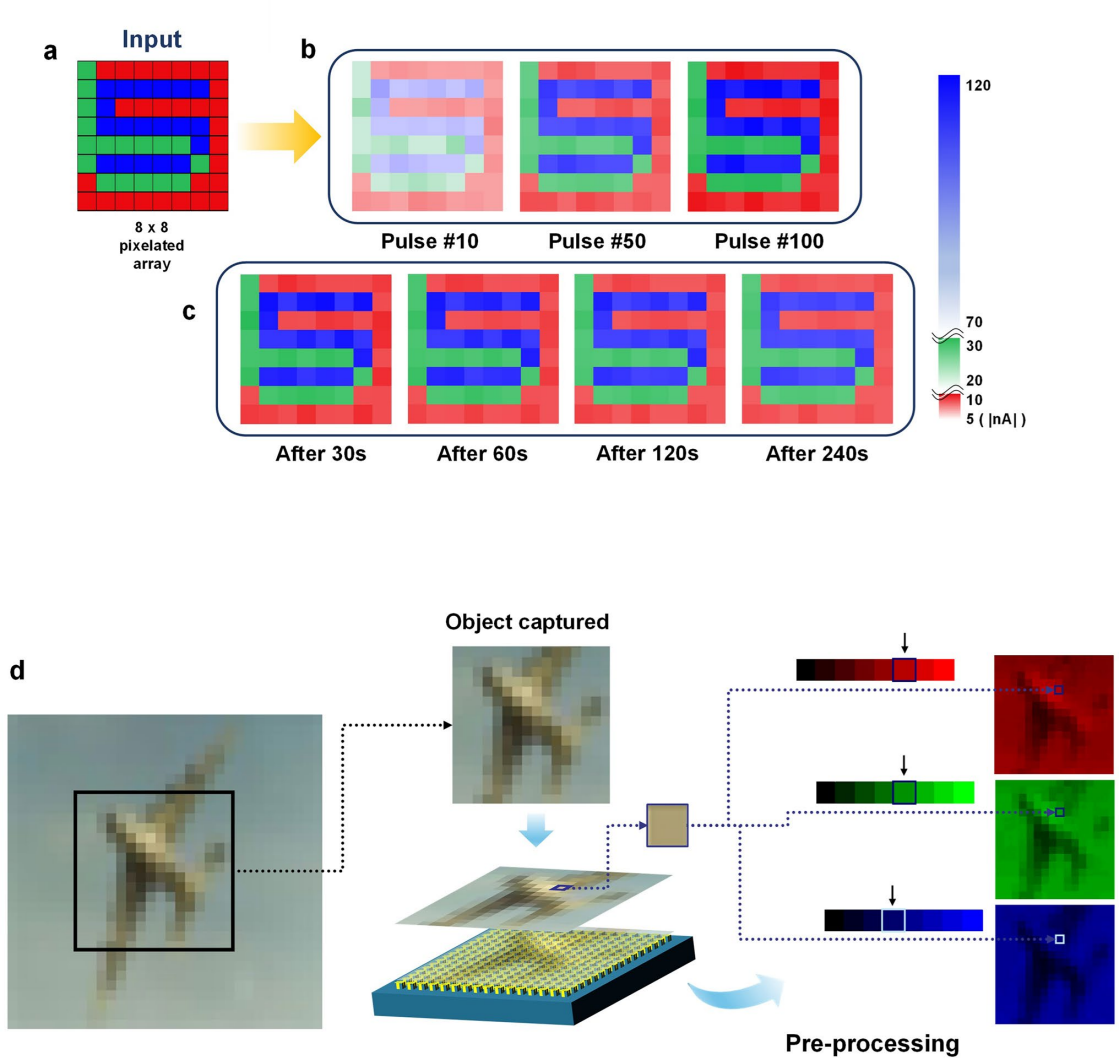
**a-c** Color contrast enhancement. 2D contour mapping of the PSCs measured on each pixel of the array at different pulse and retention time conditions. The light pulse conditions and readout voltages, *V*_GS_ and *V*_DS_, are consistent with those depicted in Fig. 6a. **d** Mixed color discrimination into three RGB channels with 3-bit resolution in the range between 1 and 40 mW cm^−2^: (B, G, R) = (0–7, 0–7, 0–7). PSC values for each state are correlated with those illustrated in Fig. 6g–i

The color discrimination ability of the CH-M-integrated device (Fig. 8d), coupled with its synaptic characteristics enhancing color contrast (Fig. 8a–c), holds great significance in the pre-processing of color images in the retina—an aspect that will be discussed in detail in the next section. Additionally, the remarkable LTP/LTD conductance tunability of the DNH-integrated transistor exhibits promising potential for efficient hardware-based CNN applications. In relation to this, the forthcoming discussion will delve into a recognition task focused on the CIFAR-10 colorful image dataset.

### Emulation of Human Visual System

**Fig. 9:**
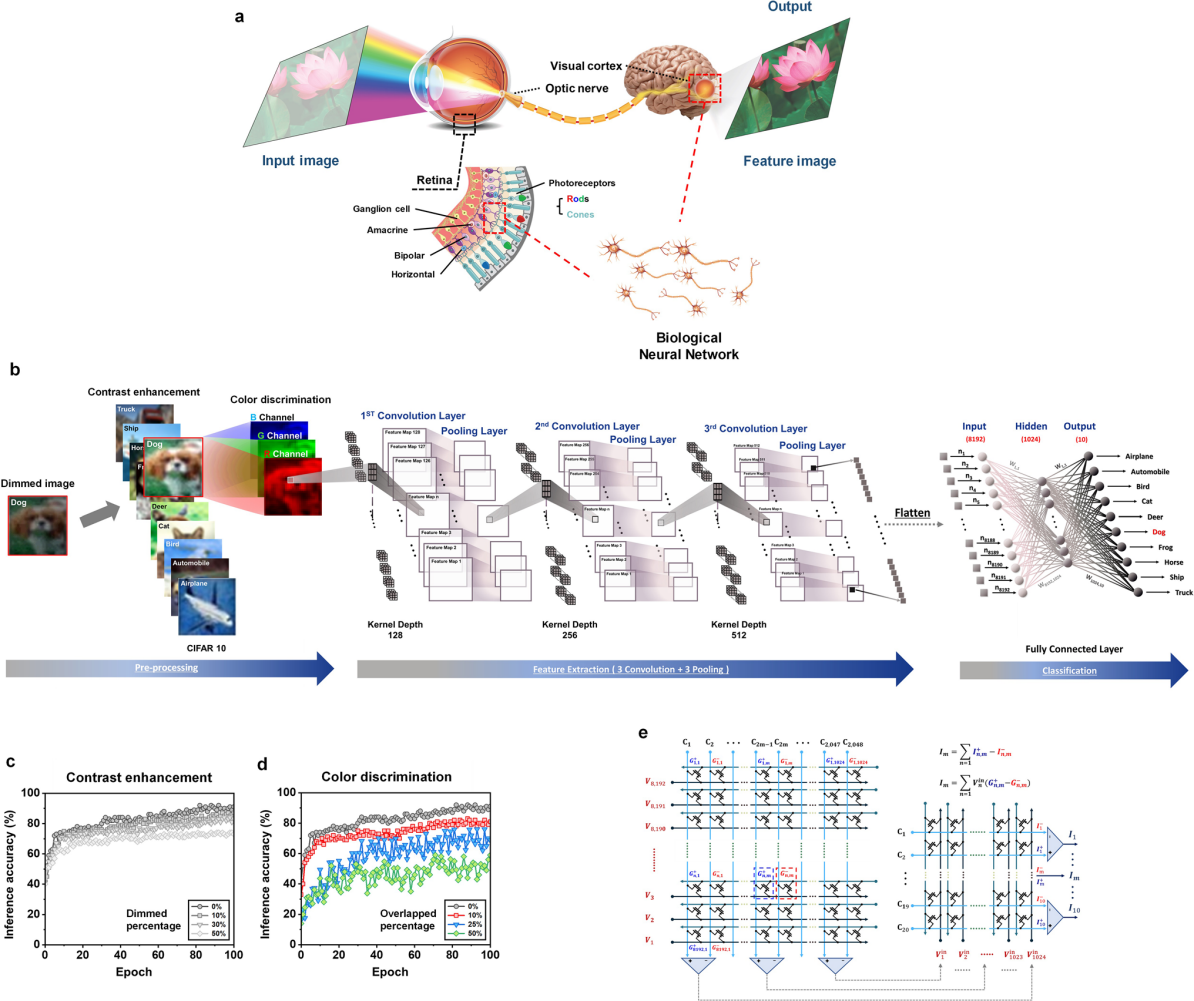
**a** Schematic diagram of the human visual recognition system comprising the retina (receiving and pretreating), optic nerve (transducing), and visual cortex (processing and memory). **b** A schematic diagram of the convolutional neural network (CNN) for color pattern image recognition, composed of pre-processed for RGB discrimination, convolutional, pooling, and fully connected layers about the CIFAR-10 dataset. **c, d** Image recognition inference accuracy of the CNN about CIFAR-10 dataset (pre-processing with 3-bit resolution RGB image, bidirectional weight update): (**c**) about color contrast enhancement function (accuracy variation depending on dimmed percentage), (**d**) about color discrimination function (accuracy variation depending on overlapped percentage). **e** Photonic synapse transistor arrays for the fully connected layers of the CNN

Figure 9a depicts a conceptual illustration of the color image recognition process in the human visual system. This system is responsible for capturing visual signals, which constitute approximately 80% of the information that humans perceive from their surroundings. In particular, the retina serves a crucial role in this visual system by not only converting light signal into electrical signals but also engaging in visual pre-processing through synaptic connections between various types of neurons, including photoreceptors, horizontal cells, bipolar cells, amacrine cells, and ganglion cells [5, 51]. This pre-processing stage effectively filters out unnecessary visual input signals, resulting in a reduction of superfluous information. Moreover, it facilitates the complex data processing within the brain, while also contributing to a decrease in power consumption [6].

To emulate the functioning of the visual system, we employ two types of photonic synapses: CH-M-integrated and DNH-integrated. The devices with CH-M, which possess color-discriminating synaptic capabilities, are utilized to design a photonic synapse array capable of detecting and pre-processing multispectral input light signals—involving a color contrast enhancement (Fig. 8a–c) and a mixed color discrimination into three RGB channels (Fig. 8d), akin to the role performed by the retina in the human eye. On the other hand, the devices integrated with DNH, which exhibit the strongest photo-synaptic characteristics as well as superior conductance tunability, are utilized in the construction of a CNN enabling high-level processing tasks such as learning and recognition (e.g., convolution kernels and fully connected layers), similar to the capabilities of the human brain (Fig. 9b).

Figure 8a–c depicts the sensing and pre-processing outcomes, specifically highlighting the color contrast enhancement, of the CH-M-integrated photonic synapse array in response to a specific light pattern. An exemplary array, configured as 8 × 8 pixels, is showcased (Fig. S22). The light pattern comprises three letters of different colors: a red-colored “3”, a green-colored “6”, and a blue-colored “5”. Notably, all letters share the same pulse conditions (power: 5 mW cm^−2^, with: 2 s, and interval: 0.3 s). By subjecting each device within a pixel to a sequence of 10 to 100 RGB light pulses, the classification of the letters becomes increasingly discernable, owing to enhanced color contrast resulting from distinct variations in channel conductance (Fig. 8b). These pre-processed output signals exhibit a non-volatile conductance change, which remains clearly distinguishable even after the removal of the pulse train (Fig. 8c). Regarding the images consisting of RGB-mixed colors with different intensity, the pre-processing step also involves separating each color input into RGB channels using the CH-M integrated transistor array before the feature extraction process through convolution. As depicted in Fig. 8d, an exemplary complex 16 × 16-pixel image, wherein each pixel is composed of RGB colors with varying intensity (3-bit resolution, 0–7), is separated into three individual RGB images through the CH-M integrated transistor array (Fig. S22). Both this mixed color discrimination and the aforementioned color contrast enhancement, emulating the pre-process in the retina, are also depicted in the early step of the artificial visual system in Fig. 9b.

The color image recognition capability of the designed artificial visual system is accessed using the DNN + NeuroSim framework [52] and the Python language for the simulator of CNN, employing the CIFAR-10 dataset. This dataset consists of color objects categorized into 10 different classes, comprising 50,000 training images and 10,000 images for inference (Fig. 9b). The color image recognition tasks are executed through a simulation approach, employing parameters derived from experimentally demonstrated photonic synapse arrays, including CH–M– and DNH-integrated photonic synapse arrays. For the recognition simulation of CIFAR-10 dataset, larger size array (32 × 32) is assumed.

The CNN stands as a powerful and widely adopted tool for image recognition [53], comprising convolution layers, pooling layers, and fully connected classification layers, as illustrated in Fig. 9b. Through the pre-processing of the CH-M-integrated photonic synapse array, the color contrast of images is enhanced, and these images are separated into three RGB channels. Subsequently, these images undergo an eight-layered CNN architecture, which includes three convolution layers—extracting high-level features that contribute to handling intricate images and attaining superior recognition accuracy—with accompanying pooling layers—reducing the spatial size of inputs—for feature extraction, as well as two fully connected layers for classification. The fully connected layer performs deep learning algorithms to accomplish image recognition tasks. The hardware transistor array that can be utilized in the two fully connected layers is illustrated in Fig. 9e. To represent both negative and positive weight values of the synapse (*W*) using the transistors that only possess positive conductance values, the synaptic weight of each cell is defined by the difference in conductance between two equivalent optoelectronic DNH devices $(W = {G}^{+}- {G}^{-})$ (Fig. S23) [54]. Moreover, an intelligent system based on optical pulse stimulation can be proposed by integrating the convolution kernels and fully connected layers with DNH-integrated photonic transistors. This system allows for the updating of the synaptic weights through utilization of light signal.

This neural network system offers notable advantages over traditional hardware neural networks that rely on electrical voltage for weight updates [12]. It achieves higher spatiotemporal resolution and implements a simpler weight-update methodology within the hardware array. Moreover, the optical programming leverages photons to provide potential benefits such as superfast speeds, low energy consumption, and a large bandwidth [55]. Based on these properties, two methods are employed to update the synaptic weights. The first method is the unidirectional approach, utilizing light-only signals, and the second method is the bidirectional approach, which enables the application of both light and voltage signals for potentiation and depression, respectively (Fig. S23b, c and Note S4) [12, 44, 56]. Detailed information regarding the feed-forward and backpropagation procedures of the CNN, as well as the specific models used to update the weight of the synapse, are explained in the Method section and Note S2.

In Figs. 9c, d and S24, the inference accuracy of the CNN for the CIFAR-10 dataset is presented. The accuracy is calculated using parameters extracted from experimentally prepared photonic synapse arrays (pre-processing: 3-bit RGB image resolution with CH-M array, convolution kernels and fully connected layers: DNH-array). Remarkably, after 100 epochs, the CNN achieves an accuracy of 92% (bidirectional, Fig. 9d) and 94% (unidirectional, Fig. S24) when processing pre-processed input images that exhibit clear discrimination among RGB regions. For reference, the superior conductance tunability of DNH-integrated synaptic device such as low nonlinearity and high symmetricity is also beneficial in achieving high accuracy. Meanwhile, when the colorful input signals are not effectively distinguished into their respective RGB channels (Figs. S25 and S26, Note S5, and Table S4), resulting in overlapping RGB components ranging from 0 to 50%, the inference accuracies decrease from 92% to 59% (bidirectional) and 94% to 64% (unidirectional). Additionally, to investigate the effect of color contrast enhancement through the pre-processing on the recognition accuracy of the system, we prepared another dimmed image dataset (50,000 for training and 10,000 for inference) that can represent the cases where the contrast is not enhanced. Figure 9c shows that the inference accuracy decreases to about 70% as the contrast of the images is dimmed to 50% (Fig. S27). These results highlight the crucial role of distinct color discrimination and color contrast enhancement in the pre-processing stage for achieving highly accurate color pattern recognition rates within the artificial visual system. Furthermore, the inference accuracy can be over 94% (bidirectional) and 95% (unidirectional) only after 60 epochs when the photonic transistors with a full 8-bit resolution RGB color-discriminating capability are utilized for the pre-processing (Fig. S28). These results suggest that implementing higher individual color resolution by optimizing the conductance gap between each state of each color is vital for achieving a sophisticated artificial visual system.

## Conclusions

The inherent capability to distinguish and enhance colors is a fundamental characteristic of the retina, enabling it to proficiently detect and pre-process visual signals effectively. Therefore, it is crucial to demonstrate photonic devices that possess multispectral color-discriminating synaptic capabilities to realize an artificial visual system. In this study, we present a mechanism that expands the photo-programming window of the floating gate-based photonic synapse transistor, by leveraging the strong induced dipole effect at excitation. This induced dipole effect at excitation is also wavelength-dependent, and thus the photo-programming window can be precisely controlled based on the wavelength of the incident light. Consequently, the synaptic device exhibits superior color discrimination capabilities.

To achieve the strong induced dipole effect under light illumination, we employ asymmetric molecules with a high intrinsic dipole moment during excitation. Particularly, this is effective when the molecule is designed to follow the ESIPT process that can provide an extended lifetime to the molecule. The degree of the induced dipole moment effect relies on the density of excited molecules, which is influenced by the spectral absorbance and wavelength-dependent excited-state lifetime of the molecule. Therefore, by utilizing ESIPT molecules to exhibit a significant spectral gradient of absorbance and excited-state lifetime, we can introduce a wavelength-dependent induced dipole moment effect to the synaptic device, resulting in remarkable color discrimination properties. Since this approach involves the modification of the floating gate layer, it can be applied to various types of channel layer-based transistors, thereby enhancing the versatility and applicability of this method. We confirm the effectiveness of this color-discriminating synaptic functionality by performing visual pre-processing on a photonic synapse-based array, thereby improving the inference accuracy of a CNN for colorful image recognition tasks over 94%.

## Supplementary Information

Below is the link to the electronic supplementary material.

Supplementary file1 (DOCX 13278 kb)
