# Supplementary material for: RGB Color-Discriminable Photonic Synapse for Neuromorphic Vision System
Source: Nanomicro Lett. 2024 Nov 29;17:78. doi: 10.1007/s40820-024-01579-y (PMC11607236; doi:10.1007/s40820-024-01579-y)
Supplement: Supplementary file 1 — Supplementary file1 (DOCX 13278 kb) [file 40820_2024_1579_MOESM1_ESM.docx]

Supporting Information for

RGB Color-Discriminable Photonic Synapse for Neuromorphic Vision System

Bum Ho Jeong^1,#^, Jaewon Lee^1,#^, Miju Ku^3,#^, Jongmin Lee^1^, Dohyung Kim^1^, Seokhyun Ham^1^, Kyu-Tae Lee^4,^*, Young-Beom Kim^3,^*, and Hui Joon Park^1,2,^*

^1^Department of Organic and Nano Engineering & Human-Tech Convergence Program, Hanyang University, Seoul 04763, Korea

^2^Department of Semiconductor Engineering, Hanyang University, Seoul 04763, Korea

^3^Department of Mechanical Engineering, Hanyang University, Seoul 04763, Korea

^4^Department of Physics, Inha University, Incheon 22212, Korea

^#^ Bum Ho Jeong, Jaewon Lee, and Miju Ku contributed equally to this work.

*Corresponding authors. E-mail: [huijoon@hanyang.ac.kr](mailto:huijoon@hanyang.ac.kr) (Hui Joon Park), [ybkim@hanyang.ac.kr](mailto:ybkim@hanyang.ac.kr) (Young-Beom Kim), [ktlee@inha.ac.kr](mailto:ktlee@inha.ac.kr) (Kyu-Tae Lee)

**Supplementary Figures, Tables and Notes**

**Note S1** **General synthesis procedure**

Synthesis procedures of DNH, DN, CH-M and C-M are described elsewhere [S1, S2].

*4-(di(naphthalen-2-yl)amino-2-(1,4,5-triphenyl-1H-imidazol-2-yl)phenol* (**DNH**): Place a mixer of 4-bromo-2-(1,4,5-triphenyl-1*H*-imidazol-2-yl)phenol (1.5 g, 3.21 mmol), di(naphthalen-2-yl)amine (1.04 g, 3.98 mmol), Pd(OAc)_2_ (0.02 g, 0.0996 mmol), P(*t*-Bu)_3_ (0.39 ml, 0.39 mmol), and *t*-BuONa (1.02 g, 10.60 mmol) in a 100 mL two necked flask under an nitrogen atmosphere. Then 40 mL of dry toluene was added. After stirring for 15 h at 110 ^o^C, the reaction mixer was cooled to room temperature. The reaction was quenched by water and extracted with ethyl acetate. The organic layer was collected and concentrated. The residue was purified via column chromatography by using hexane/dichloromethane (80:20 v/v) as eluent to afford yellow solid (1.85 g, 88%).

*N-(naphthalen-2-yl)-N-(3-(1,4,5-triphenyl-1H-imidazol-2-yl)phenyl)naphthalen-2-amine* (**DN**): Place a mixer of 2-(3-bromophenyl)-1,4,5-triphenyl-1*H*-imidazole (1.50 g, 3.32 mmol), di(naphthalen-2-yl)amine (1.07 g, 3.98 mmol), Pd(OAC)_2_ (0.02 g, 0.997 mmol), P(*t*-Bu)_3_ (0.40 mL, 0.40 mmol), and *t*-BuONa (1.05 g, 10.96 mmol) in a 100 mL two necked flask under an nitrogen atmosphere. Then 40 mL of dry toluene was added. After stirring for 15 h at 110 ^o^C, the reaction mixer was cooled to room temperature. The reaction was quenched by water and extracted with ethyl acetate. The organic layer was collected and concentrated. The residue was purified via column chromatography by using hexane/dichloromethane (80:20 v/v) as eluent to afford white solid (1.74 g, 82%).

*(E)-3-(4-(dimethylamino)phenyl)-1-(1-hydroxynaphtalen-2-yl)prop-2-en-1-one* (**CH-M**): 1.50 g (8.06 mmol) of 2-Acetyl-1-naphthol and 1.20 g of 4-(dimethylamino)benzaldehyde 8.06 mmol) were placed in 100 mL round-bottom flask and dissolved in 30 mL of ethanol. 0.662 mL of pyrrolidine was added to the solution and stirred overnight at room temperature. The color of solution turns red from yellow after the addition of pyrrolidine. After the completion of the reaction, the mixture was poured into water, filtered, and washed with cooled ethanol. The filtrate was dried using MgSO_4_, and the crude product was column-purified by silica-gel column chromatography using EA and n-hexane as eluent (25:75 v/v). 1.93 g of product was obtained after the purification (Y=64.5%).

*(E)-3-(4-(dimethylamino)phenyl)-1-(naphthalen-2-yl)prop-2-en-1-one* (**C-M**): 2-Acetylnapthalene (0.50 g, 2.93 mmol) and 4-dimethylamino benzaldehyde (0.44 g, 2.93 mmol) were placed in 100ml round bottom flask and charged with 10 mL of ethanol. 0.21 g (2.93 mmol) of pyrrolidine was added to the solution and stirred overnight at room temperature. After completion of the reaction, the solvent was removed under reduced pressure, the residue was dissolved in ethyl acetate and washed with water. The organic layer was separated and dried over Na_2_SO_4_. The organic layer was evaporated in vacuum and the crude product was purified by column chromatography (Hexane/EtOAc = 10:1) to give C-M (600 mg) as yellow solid in 66% yield.

**Fig. S1 a-c** UPS binding energy profiles showing the secondary electron cut-off energy (*E*_cut-off_), associated with the work functions, and the valence band maximum (VBM). **(a)** CH-M, **(b)** C8-BTBT, and **(c)** Al_2_O_3_ thin films. **d** Tauc plots of CH-M and C8-BTBT thin film. **e** Energy band diagram of the components composing photonic synapse transistor (before contact) showing the VBM (obtained from UPS data), conduction band minimum (CBM) (calculated from the UPS data and optical bandgap derived from the Tauc plots), and Fermi energy level (from the UPS data)

**Fig. S2** Four-level photocycle of the ESIPT process (DNH: UV-responsive molecule, and CH-M: visible light-responsive molecule)

**Fig. S3 a** Energy-band diagrams of the components composing photonic synapse transistor (before contact). **b** Schematic version of (a) without dipole effect. **c** With induced dipole effect. **d** With maximized induced dipole effect at the excitation. *V*_b_ and *V*_d_ represent built-in potential and increased built-in potential with induced dipole effect

**Fig. S4** Orbital diagrams of organic molecules for floating gate, estimated by DFT. **a** DNH, **b** DN, **c** CH-M, and **d** C-M. **(a, c)** ESIPT-molecules and **(b, d)** non-ESIPT-molecule. Theoretically calculated dipole moment values and energy band levels are included

**Fig. S5** UV-visible absorbance spectra of organic semiconductor films casted on quartz substrates under various light irradiation times from 0 min (fresh) up to 30 min: **a** DNH and **b** CH-M


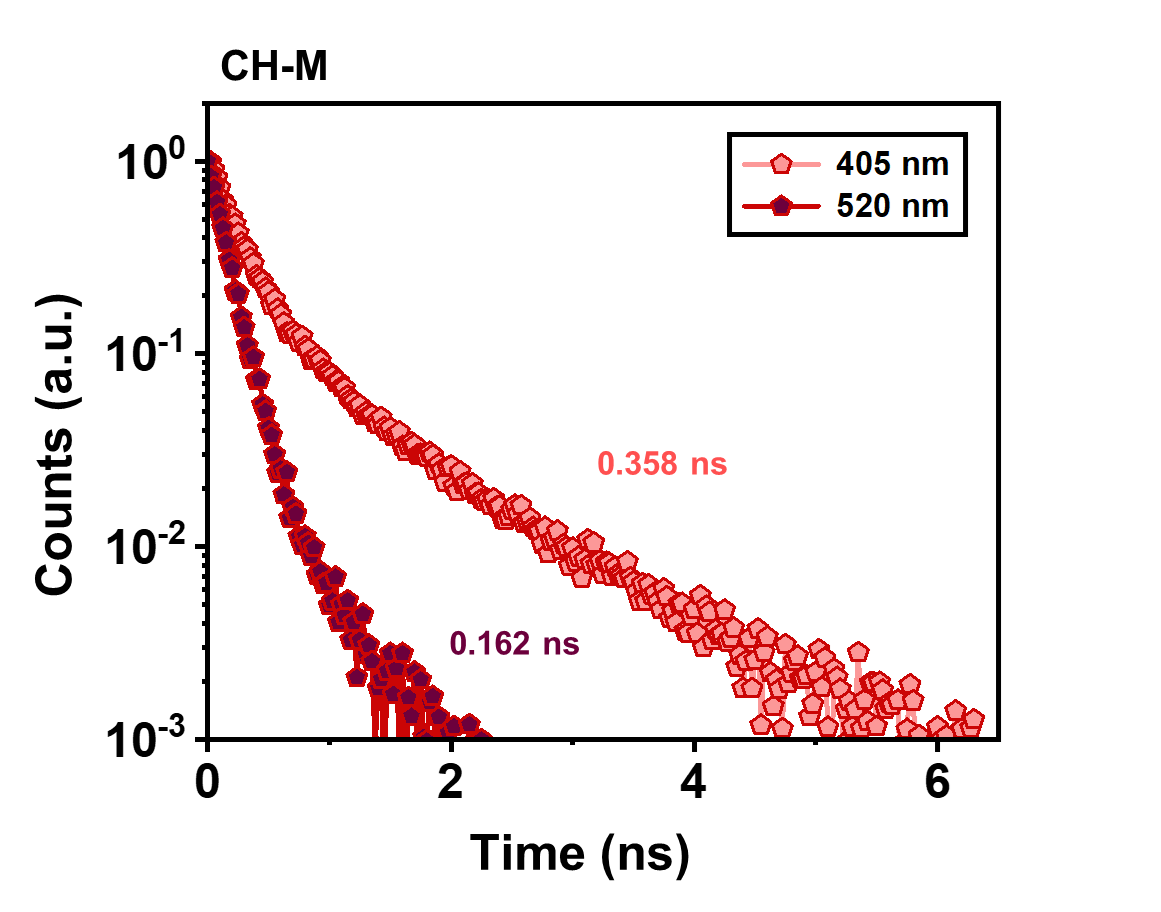


**Fig. S6** Time-resolved photoluminescence (TRPL) of CH-M thin film casted on quartz excited at 405 and 520 nm wavelengths (*τ*_avg_: 3.58 ×10^-10^ s at 405 nm, 1.62 ×10^-10^ s at 520 nm)

**Fig. S7** Spectral distribution of R, G, B and UV light sources, with center wavelengths of 630 nm, 525 nm, 450 nm, and 365 nm, respectively.

**Fig. S8** Output characteristics of photonic synapse transistors under both dark and light illumination conditions (light power: 5mWcm^-2^), with different *V*_G_ values. **a** DNH (left panel) and DN (right panel) at dark. **b** CH-M (left panel) and C-M (right panel) at dark. **c** DNH (left panel) and DN (right panel) under UV-light illumination (365 nm). **d** CH-M (left panel) and C-M (right panel) under blue-light illumination (450 nm)

**Fig. S9 a** Transfer curve variation of the C-M-integrated photonic synapse transistor under light illumination (450 nm blue, 525 nm green, and 630 nm red at 5 mWcm^-2^). **b** Temporal changes in *I*_D_ of the C-M-integrated photonic synapse transistor under 10 s light illumination (450 nm blue, 525 nm green, and 630 nm red at 5 mWcm^-2^) at *V*_D_ = -10 V and *V*_G_ = -4 V

**Fig. S10** Transfer characteristics (*I*_DS_ vs *V*_GS_) under dark and 10 s light illumination at *V*_DS_ = -10 V for devices. **a** without organic floating gate layer, **b** with DNH, and **c** with CH-M

**Fig. S11** Transfer curves of photonic synapse transistors at various temperatures up to 130°C about photo-programming and electrical erasing operations. **a** DNH (photo-programming pulse conditions: 365 nm UV light, 5 mWcm^-2^, and width of 10 s / electrical-programming pulse conditions: *V*_G_ = -60 V and width of 1 s). **b** CH-M (photo-programming pulse conditions: 450 nm blue light, 5 mWcm^-2^, and width of 10 s / electrical-programming pulse conditions: *V*_G_ = -60 V and width of 1 s)

**Fig. S12** EPSC behaviors of photonic synapses depending on the light pulse intensity. **a** DNH (365 nm UV light with 2 s pulse width) at *V*_DS_ = -10 V and *V*_G_ = -1 V. **b** CH-M (450 nm blue light with 2 s pulse width) at *V*_DS_ = -10 V and *V*_G_ = -4 V

**Fig. S13** Energy consumption values for reading. **a** 15.05 fJ for DNH at 365 nm UV light pulse. **b** 14.37 fJ for CH-M at 450 nm blue light pulse


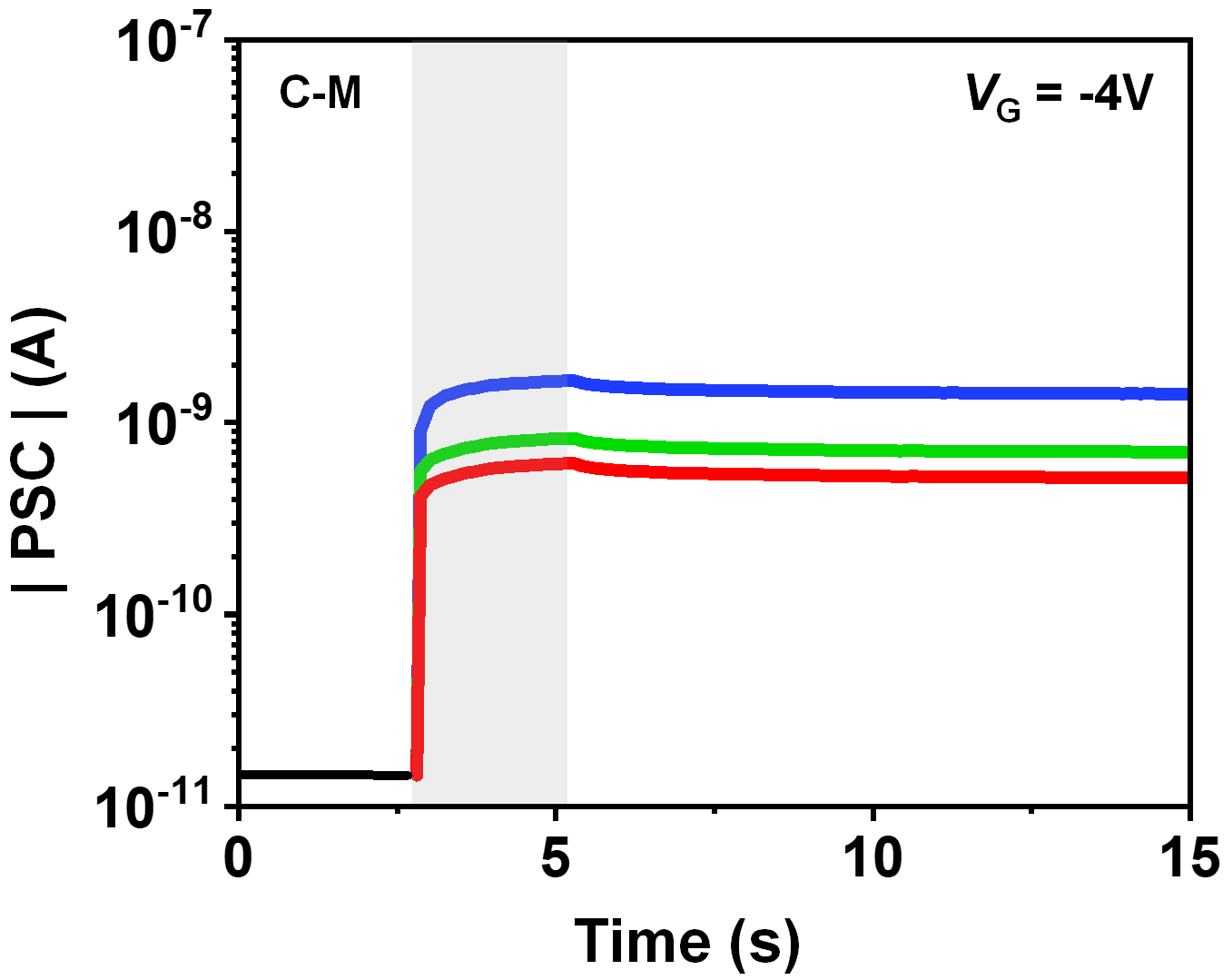


**Fig. S14** EPSC behavior of the C-M-integrated photonic synapse under light illumination (450 nm blue, 525 nm green, and 630 nm red at 5 mWcm^-2^ with 2 s pulse width) at *V*_DS_ = -10 V and *V*_G_ = -4 V

**Fig. S15** PPF index (A_2_/A_1_) of CH-M-integrated photonic synapse as a function of interval between two consecutive light pulses (0.1 s ≤ ∆t ≤ 16 s) at *V*_G_ = -4 V and *V*_D_ = -10 V. **a** 525 nm green light pulses. **b** 630 nm red light pulses


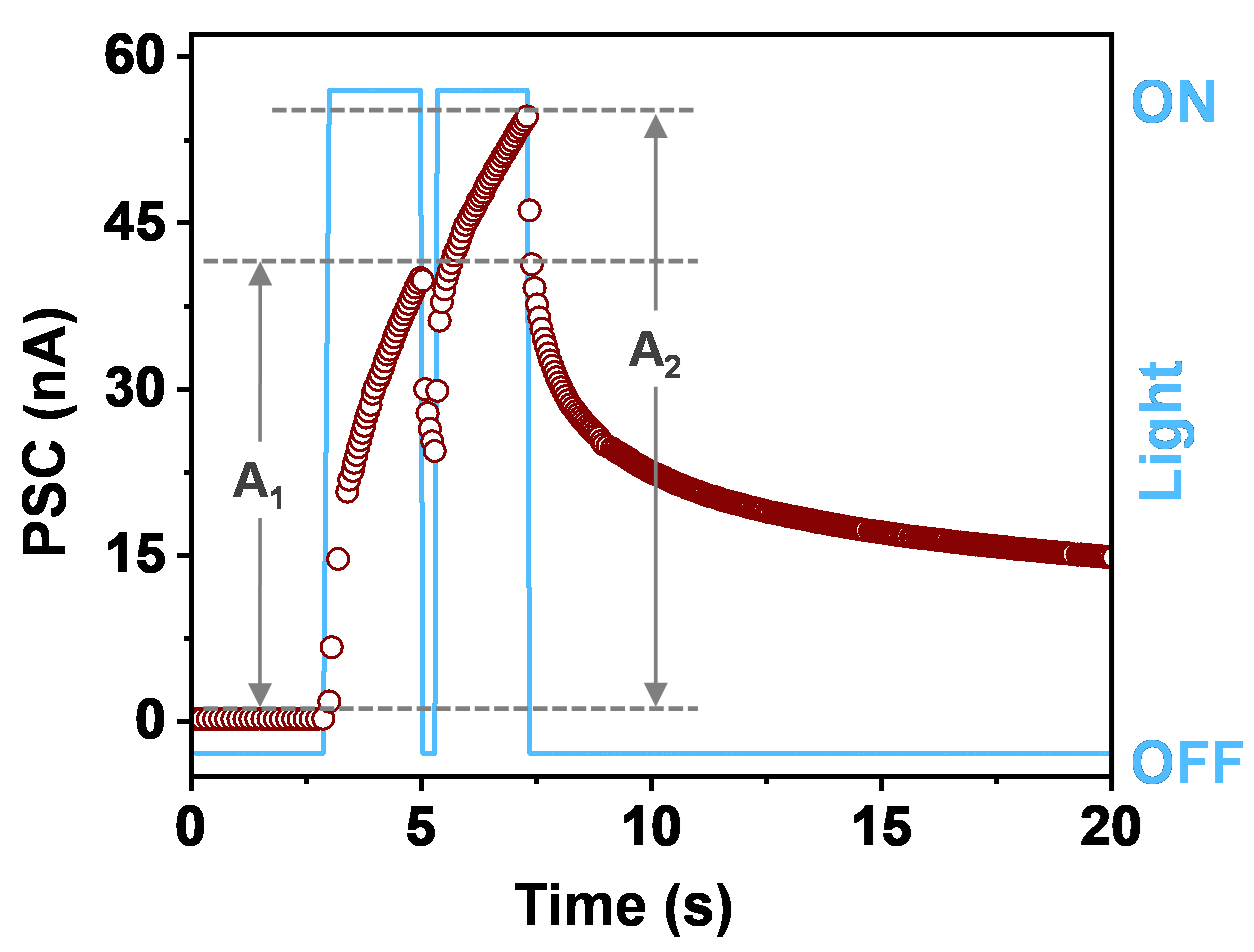


**Fig. S16** An exemplary PPF behavior of the DNH-integrated photonic synapse at a pair of consecutive optical pulses (UV light pulses with 2 s width and 0.3 s interval) at *V*_G_ = -1 V and *V*_D_ = -10 V

**Fig. S17 a** LTP/LTD behavior of the DNH-integrated photonic transistor about 365 nm UV light. **b** LTP/LTD behavior of the DN-integrated photonic transistor about 365 nm UV light. **c-e** LTP/LTD behavior of the CH-M based transistor about **(c)** 450 nm blue light, **(d)** 525 nm green light, and **(e)** 630 nm red light. The non-linearity coefficients (1/*A*_P_ and 1/*A*_D_) estimated from LTP/LTD curves using the equations in the following Supplementary Note 1 are 1/4.38 and 1/4.05 for DNH, 1/3.81 and 1/2.43 for DN, 1/0.49 and 1/0.32 for CH-M at blue, 1/0.48 and 1/0.35 for CH-M at green, and 1/2.482 and 1/1.556 for CH-M at red. The A value is inversely proportional to nonlinearity of the LTP/LTD curves

**Note S2** **Estimating method of nonlinearity values by calculation of LTP/LTD curves**

For simulating the image recognition tasks of the CNN about the CIFAR-10 dataset using the NeuroSim simulator, the nonlinearity of the LTP and LTD characteristics curves are extracted by modeling the conductance update behaviors of the device by the following conductance update equations [S3]:

$G_{\mathrm{LTP}}=G_{\min}+B\left( 1-e^{\frac{P}{A}} \right)$ (S1)

$G_{\mathrm{LTD}}=G_{\max}-B\left( 1-e^{\frac{P-P\max}{A}} \right)$ (S2)

$B=\frac{G_{\max}-G_{\min}}{1-e^{-\frac{P_{\max}}{A}}}$ (S3)

where *G*_LTP_ and *G*_LTD_ are the conductance values of device for potentiation and depression, *G*_max_ and *G*_min_ represents maximum and minimum conductance values at each the potentiation and depression steps, *P* is the pulse number, and *P*_max_ is the maximum value related to step size of the weight update. The *A* value is inversely proportional to the nonlinearity of the LTP/LTD curve, and *B* is the fitting parameter as a function of *A*.

Depending on nonlinearity (1/*A*) from 0 to 10, the variation in the LTP/LTD curves are shown in Fig. S18. As the nonlinearity (1/*A*) values increases from zero, both LTP and LTD curves become more non-linear.

**Fig. S18** **a, b** Theoretically estimated LTP (left) and LTD (right) curves by conductance update equations (1~3) in Supplementary Note 1: **(a)** LTP and **(b)** LTD with nonlinearity values between 0 and 10

**Fig. S19** Retention capabilities of photo-programmed and electrically erased states of the ESIPT-based photonic synapse in an ambient condition. **a** Photo-programming pulse conditions of DNH-case: 365 nm UV light, 5 mWcm^-2^, and width of 10 s / electrical-programming pulse conditions of DNH-case: *V*_G_ = -60 V and width of 1 s. **b** Photo-programming pulse conditions of CH-M-case: 450 nm blue light, 5 mWcm^-2^, and width of 10 s / electrical-programming pulse conditions of CH-M-case: *V*_G_ = -60 V and width of 1 s

**Fig. S20** Photosynaptic characteristics of the CH-M-integrated photonic synapse under respective RGB-light pulses with various light intensities (e.g., <1 mWcm^-2^). The consecutive light pulses conditions (R: 630 nm, G: 525 nm, and B: 450 nm) are set to a width of 2 s, an interval of 1.0 s, a spike number of 20, and intensities from 0.05 to 1 mWcm^-2^, respectively.

**Note S3 RGB-mixed color discrimination (resolution of the color-discrimination capability for the pre-processing of CIFAR-10 colorful images)**

For validating color image processibility of CH-M-integrated photonic synapse about the CIFAR-10 dataset with 3-bit resolution individual RGB colors, the following three steps are presented.

**Step 1.** The extent of PSC and conductance change should be perfectly distinguished without any duplication for each RGB primary colors, as shown in Fig. 5a-f.

**Step 2.** The difference in PSC values (and the corresponding conductance) between each state of B color must be significantly larger than the highest PSC value of the states of the G and R colors. Moreover, the difference in PSC value between each state of G color should be larger than the highest PSC value among states in R color for a clear pre-processing of RGB color images.

An illustrative example of a 3-bit resolution RGB color-discrimination case is presented in the Fig. S21a-c, based on the performance characteristics of the CH-M-integrated photonic synapse. First of all, as mentioned above step 1, the extent of PSC and conductance change of each RGB color is perfectly distinguished without any duplication. Then, the difference in PSC values of each state of the blue color (29.0 nA) is notably larger than the highest PSC value of the states of the green (22.2 nA) and red (1.75 nA) colors. Similarly, the difference in PSC value of each state of the green color (2.75 nA) is considerably larger than the highest PSC value of the states of the red color (1.75 nA). Under the specific given conditions, our designed CH-M-integrated photonic synapse have a capability to separate a 3-bit resolution color signal within the range of (R, G, B) = (0~7, 0~7, 0~7).

**Step 3.** For the aforementioned color-discrimination to be valid, the deviation of PSC value for B color at each state must be considerably smaller than the gap between the states for G and R colors. Likewise, the deviation of PSC value for G color at each state should also be smaller than the gap between the states of R color. The calculated standard deviation values of RGB colors are presented in Fig. S21a-c.

The deviation of the PSC value for B color at each state (*σ* = | 1.048 × 10^-3^ | nA) is significantly smaller than the interval between the states of G color (2.75 nA) and R (0.205 nA) color. In the same way, the deviation of PSC value for G color at each state (*σ* = | 7.379 × 10^-5^ | nA) is also remarkably smaller than the interval between the states of R color (0.205 nA).

Based on the superior RGB color discrimination functionality of the CH-M-integrated photonic synapse (3-bit resolution individual RGB colors), we believe that this approach paves a way for the realization of an artificial visual system.

**Fig. S21** UV-visible absorbance spectra and Tauc plots showing the optical bandgap energy (*E*_G_) of organic semiconductor films casted on quartz: **a** P3HT and **b** PTB7


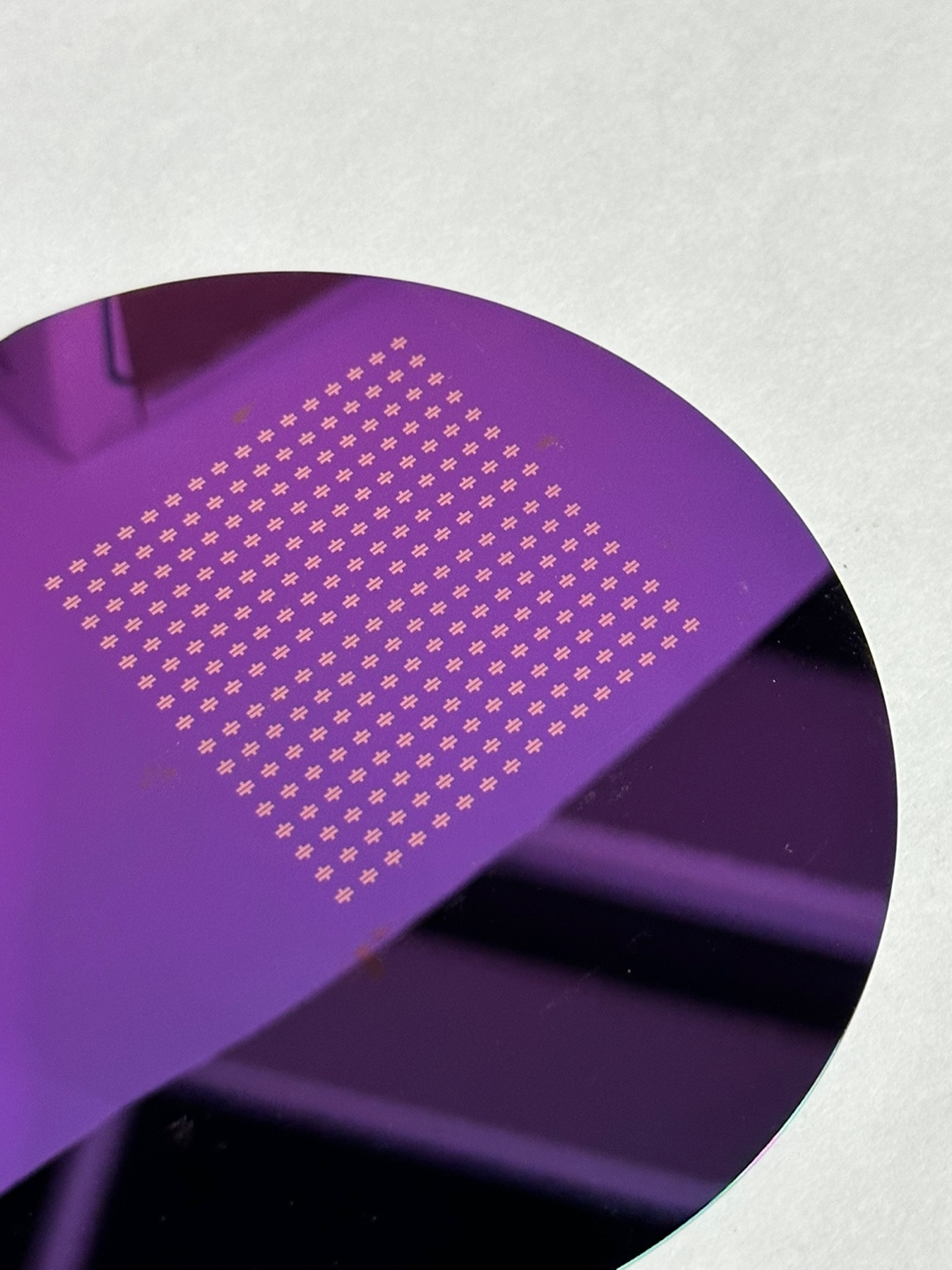


**Fig. S22** 16 × 16 photonic synapse array

**Note S4 Unidirectional and bidirectional weight update method**

In the unidirectional weight update method, *G*^+^ and *G*^-^ only increases for weight potentiation and weight depression, respectively: weight potentiation $(\boldsymbol{W}\boldsymbol{\uparrow})=\boldsymbol{G}^{\boldsymbol{+}}\boldsymbol{\uparrow} - G^{-}$ and weight depression: $\boldsymbol{W}\boldsymbol{\downarrow} =G^{+} - \boldsymbol{G}^{\boldsymbol{-}}\boldsymbol{\uparrow}$. Simply put, for potentiation, the conductance of one of the two transistors, representing *G*^+^, increases, and, for depression, that of one of the two transistors, representing *G*^–^, increases. In this case, the device only requires conductance increase behavior, which can be achieved through light pulse-based mechanism. When the conductance *G* of the device reaches *G*_max_, it undergoes the dynamics of refresh and reprogram, as depicted in Fig. S23b.

In the bidirectional weight update method, *G*^+^ increase or decrease for weight potentiation or weight depression, respectively. Similarly, *G*^-^ increases or decreases for weight depression or weight potentiation, respectively: weight potentiation ($\boldsymbol{W}\boldsymbol{\uparrow})=\boldsymbol{G}^{\boldsymbol{+}}\boldsymbol{\uparrow} - \boldsymbol{G}^{\boldsymbol{-}}\boldsymbol{\downarrow}$ and depression: $\boldsymbol{W}\boldsymbol{\downarrow} =\boldsymbol{G}^{\boldsymbol{+}}\boldsymbol{\downarrow}- \boldsymbol{G}^{\boldsymbol{-}}\boldsymbol{\uparrow}$. In essence, for potentiation, the conductance of one of the two transistors, representing *G*^+^, increases, while that of the other transistor, representing *G*^–^, decreases. Conversely, for depression, the conductance of one of the two transistors, representing *G*^+^, decreases, while that of the other transistor, representing *G*^–^, increases. In this case, a combination of light pulse-based conductance increase behavior and voltage-based conductance decrease behavior is employed for conductance variation, as depicted in Fig. S23c.

**Fig. S23 a** Representation of synaptic weight using the conductance difference between two equivalent photonic artificial synapse devices. **b**, **c** Detailed weight updating processing based on operations of weight dynamics for the optoelectronic synapse devices: **(b)** Unidirectional update method. **(c)** Bidirectional update method


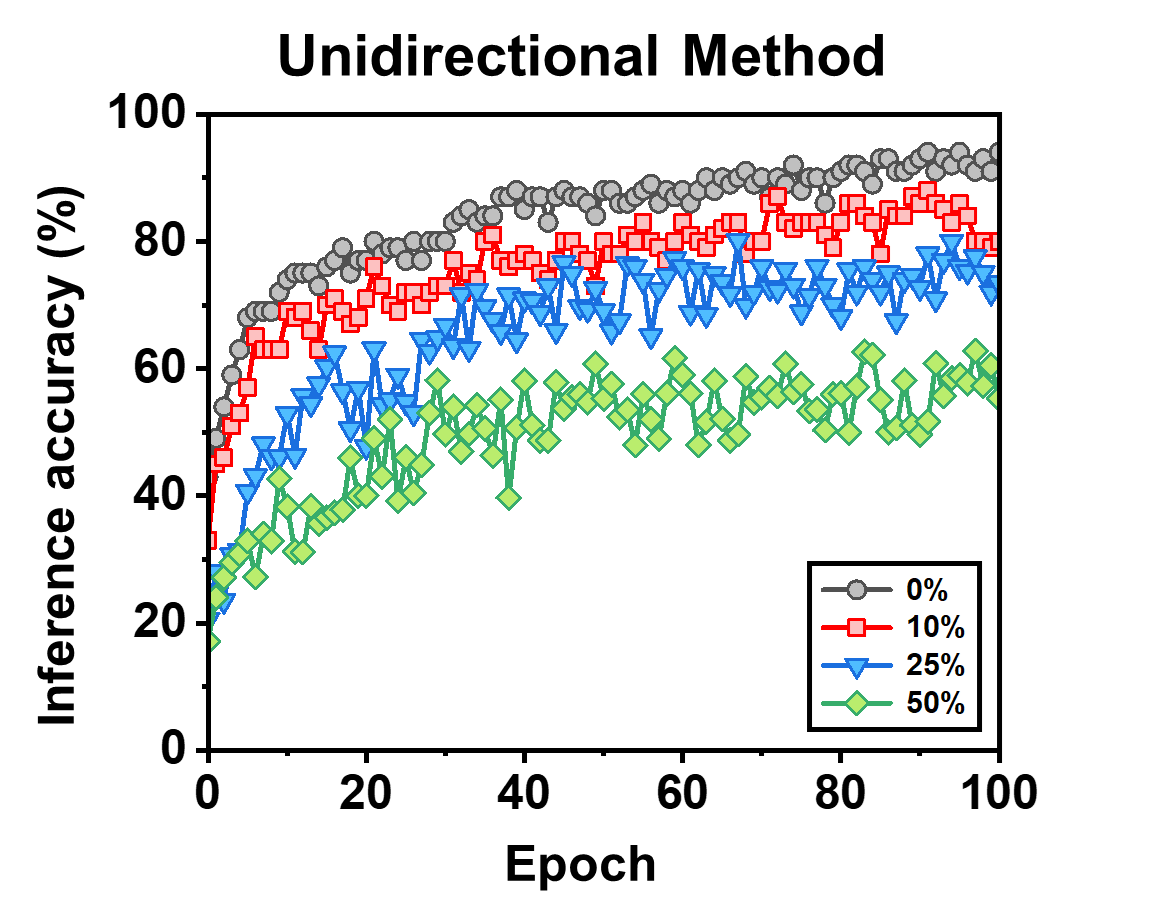


**Fig. S24** Image recognition inference accuracy of the CNN about CIFAR-10 dataset (pre-processing with 3-bit resolution RGB image, unidirectional weight update): about color discrimination function (accuracy variation depending on overlapped percentage)

**Note S5** **Method to produce RGB channel images with overlapped region**

In this work, the CH-M-integrated photonic synapse exhibits 3-bit color discrimination capability. Therefore, we normalize the color intensity values of each pixel from CIFAR-10 8-bit images, which originally range from 0 to 255, to 3-bit images with values in the range of 0 to 7 (Fig. S25a, c). The pixel values are then separated into RGB channels (Fig. S25b, d). To incorporate overlapped components of RGB colors, specific portions of the image are designed as overlapped regions, such as 10% (intensity: 0.8), 25% (intensity: 2.0), and 50% (intensity: 4.0) (Fig. S25b, d). The results of setting overlapping region is presented in Fig.S25e.

When the values of each pixel in the RGB channel reach the lower limit of overlapped region (L.L.) or do not reach the higher limit of overlapped region (H.L.), we adjust the values according to a specific rule, as explained in Table S3 to implement the overlapping RGB component. In the red channel, there is no adjustment to the pixel values. However, in the green channel, the pixel values are adjusted based on whether there is an overlapped component between green channel and red channels. If there is an overlapped component, the values of each pixel in the green channel are modified to a much lower value. The same procedure is applied to pixel values in the blue channel. This method enables the preparation of a pre-processed image for a convolutional neural network (CNN) simulation, reflecting the overlapping RGB components perceived by the device when processing a color image.

**Fig. S25** Pre-processed images of a randomly selected “DOG” input image in CIFAR-10 dataset [3-bit resolution: (R,G,B) = (0~7, 0~7, 0~7)]. The color input image is separated into RGB three channels, where the overlapped RGB components reach 0, 10, 25, and 50%

**Fig. S26** Illustration of the creating pre-processed image. **a, c** Normalizing color intensity values of 8-bit image pixels to 3-bit image pixels. **b, d** Separation into RGB three channels without (0%) and with overlapped portions (10%). **e** Lower limit and higher limit of overlapped region in each channel


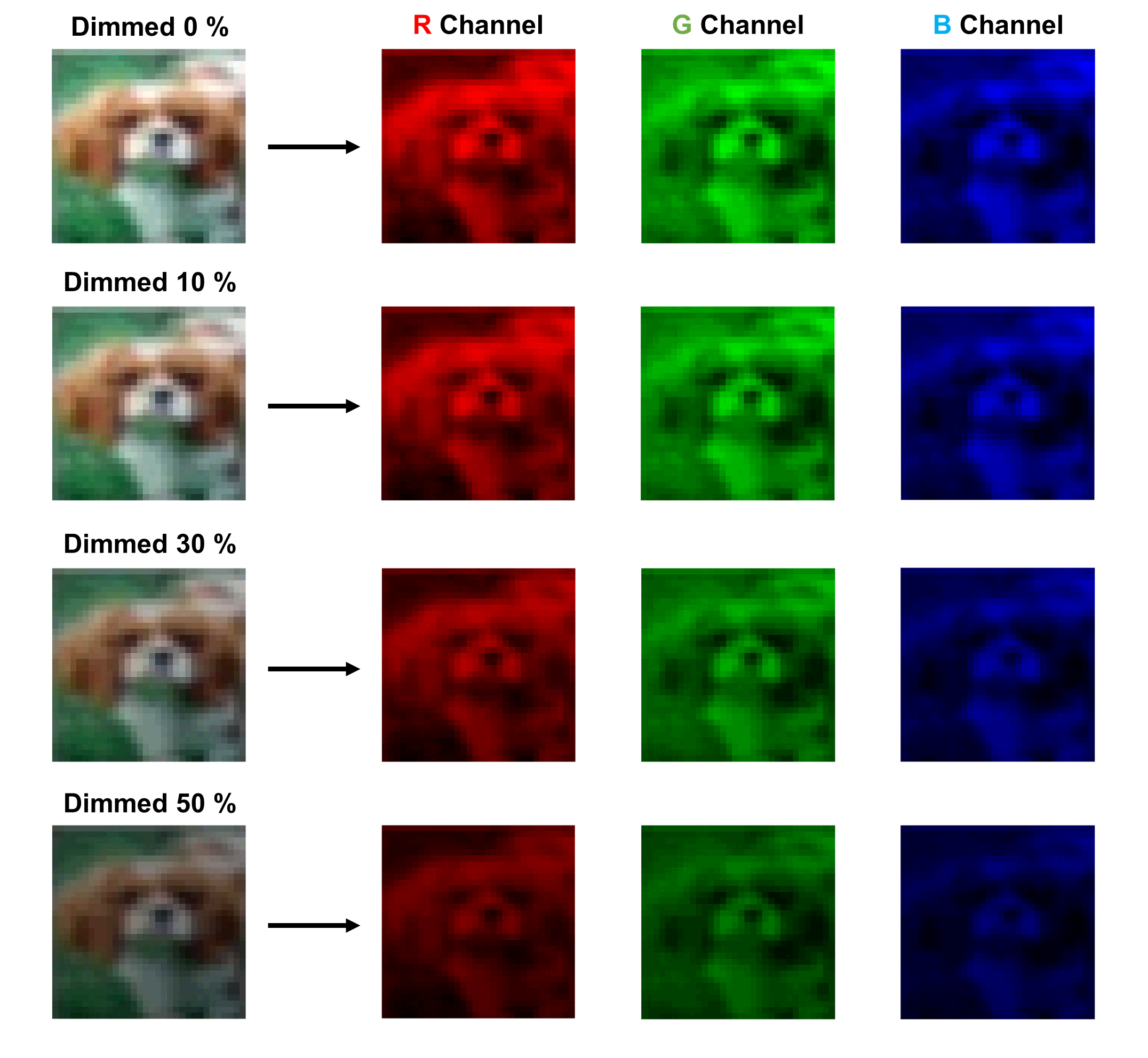


**Fig. S27** Pre-processed images of a randomly selected “DOG” input image in CIFAR-10 dataset [3-bit resolution: (R,G,B) = (0~7, 0~7, 0~7)]. The color contrast of the image is adjusted by dimming the image by 0, 10, 25, and 50 %

**Fig. S28** Image recognition inference accuracy of the CNN about CIFAR-10 dataset after pre-processing utilizing the photonic synapse flash memory device with a full 8-bit resolution RGB color-discrimination capability. **a** Bidirectional, **b** Unidirectional weight update

**Table S1** Fitted parameters of TRPL decay curves of organic molecule thin films (266 nm wavelength excitation: DNH and DN / 405 nm wavelength excitation: CH-M, and C-M)

|  | $\boldsymbol{A}_{\boldsymbol{1}}$**(%)** | $\boldsymbol{\tau}_{\boldsymbol{1}}$**(ns)** | $\boldsymbol{A}_{\boldsymbol{2}}$**(%)** | $\boldsymbol{\tau}_{\boldsymbol{2}}$**(ns)** | $\boldsymbol{\tau}_{\mathbf{avg}}$**(ns)** |
| --- | --- | --- | --- | --- | --- |
| **DNH** | 70.90 | 0.351 | 29.10 | 1.740 | 0.755 |
| **DN** | 76.18 | 0.272 | 23.82 | 1.305 | 0.518 |
| **CH-M** | 83.32 | 0.223 | 16.68 | 1.073 | 0.365 |
| **C-M** | 98.72 | 0.162 | 1.28 | 0.994 | 0.173 |

where Counts(*t*) $=A_{1}\cdot\exp\left( -\frac{t}{\tau_{1}} \right)+A_{2}\cdot\exp\left( -\frac{t}{\tau_{2}} \right)$

$\tau_{\mathrm{avg}}$: amplitude weighted average lifetime

**Table S2** Fitted parameters of PPF index

|  | $\boldsymbol{C}_{\boldsymbol{1}}$ | $\boldsymbol{\tau}_{\boldsymbol{1}}$**(s)** | $\boldsymbol{C}_{\boldsymbol{2}}$ | $\boldsymbol{\tau}_{\boldsymbol{2}}$**(s)** |
| --- | --- | --- | --- | --- |
| **DNH (UV)** | 0.27 | 0.97 | 0.73 | 218.24 |
| **CH-M (B)** | 0.13 | 1.34 | 0.87 | 206.87 |
| **CH-M (G)** | 0.14 | 1.25 | 0.86 | 149.05 |
| **CH-M (R)** | 0.06 | 1.17 | 0.94 | 130.96 |

where PPF index $=C_{1}\cdot\exp\left( -\frac{\Delta t}{\tau_{1}} \right)+C_{2}\cdot\exp\left( -\frac{\Delta t}{\tau_{2}} \right)$

**Table S3** 3-bit resolution mixed RGB color-discrimination (data for Fig. 6g-i)

| **Power**  mWcm^-2^ |  | **Blue** | | |  | **Green** | | |  | **Red** | | |
| --- | --- | --- | --- | --- | --- | --- | --- | --- | --- | --- | --- | --- |
|  |  | State | PSC after  100^th^ pulse  (\|nA\|) | Standard  deviation  (\|nA\|) |  | State | PSC after  100^th^ pulse  (\|nA\|) | Standard  deviation  (\|nA\|) |  | State | PSC after  100^th^ pulse  (\|nA\|) | Standard  deviation  (\|nA\|) |
| 40 |  | 7 | 270 | ±1.048  × 10^-3^ |  | 7 | 22.00 | ±7.379  ×10^-5^ |  | 7 | 1.750 | ±4.538  ×10^-6^ |
| 30 |  | 6 | 241 | ±9.354  × 10^-4^ |  | 6 | 19.25 | ±6.457  ×10^-5^ |  | 6 | 1.545 | ±4.006  ×10^-6^ |
| 20 |  | 5 | 212 | ±8.293  × 10^-4^ |  | 5 | 16.50 | ±4.989  ×10^-5^ |  | 5 | 1.340 | ±3.289  ×10^-6^ |
| 10 |  | 4 | 183 | ±6.458  × 10^-4^ |  | 4 | 13.75 | ±3.374  ×10^-5^ |  | 4 | 1.135 | ±3.030  ×10^-6^ |
| 5 |  | 3 | 154 | ±6.375  × 10^-4^ |  | 3 | 11.00 | ±3.135  ×10^-5^ |  | 3 | 0.930 | ±2.621  ×10^-6^ |
| 3 |  | 2 | 125 | ±3.920  × 10^-4^ |  | 2 | 8.25 | ±2.381  ×10^-5^ |  | 2 | 0.725 | ±1.891  ×10^-6^ |
| 1 |  | 1 | 96 | ±3.560  × 10^-4^ |  | 1 | 5.50 | ±1.823  ×10^-5^ |  | 1 | 0.520 | ±1.581  ×10^-6^ |
| 0 |  | 0 | 0 | 0 |  | 0 | 0 | 0 |  | 0 | 0 | 0 |

**Table S4** Table. Rule of modifying pixel values for each RGB channel to produce RGB overlapped images

| Results | |
| --- | --- |
| A intensity of pixel | (I_red.1_, I_green.1_, I_blue.1_) → (I_red.2_, I_green.2_, I_blue.2_) |
| Red channel pixel values | |
| Rule | No adjustment to values in the red channel whether the value is over 90% or not. |
| Case 1 | I_red.2_ = I_red.1_ |
| Green channel pixel values | |
| Rule | Adjustment to values in the green channel based on values of red channel. |
| Case 1 | I_red_ > L.L. of red channel, I_green_ < H.L. of green channel  → I_green.2_ = H.L. - (7 - I_green.1_) |
| Case 2 | I_red_ > L.L of green channel, I_green_ > H.L. of green channel  → I_green.2_ = I_green.1_ |
| Blue channel pixel values | |
| Rule | Adjustment to values in the blue channel based on values of green channel. |
| Case 1 | Case 1. I_green_ > L.L. of green channel, I_blue_ < H.L. of blue channel  → I_blue.2_ = H.L. - (7 - I_blue.1_) |
| Case 2 | Case 2. I_green_ > L.L. of green channel, I_blue_ > H.L. of blue channel  → I_blue.2_ = I_blue.1_ |

**Supplementary References**

[S1] B. Jo, H. Park, E. Kamaraj, S. Lee, B. Jung et al., Synergistic Effect of Excited State Property and Aggregation Characteristics of Organic Semiconductor on Efficient Hole-Transportation in Perovskite Device. Adv. Funct. Mater. **31**, 2007180 (2021). <https://doi.org/10.1002/adfm.202007180>

[S2] J. Lee, B. H. Jeong, E. Kamaraj, D. Kim, H. Kim et al., Light-enhanced molecular polarity enabling multispectral color-cognitive memristor for neuromorphic visual system. Nat. Commun. **14**, 5775 (2023). <https://doi.org/10.1038/s41467-023-41419-y>

[S3] P. -Y Chen, X. Peng, S. Yu, NeuroSim: A circuit-level macro model for benchmarking neuro-inspired architectures in online learning. IEEE Trans. Comput. -Aided Des. Integr. Circuits Syst. **37**, 3067 (2018). <https://doi.org/10.1109/TCAD.2018.2789723>
